# NAD+‐Boosters Improve Mitochondria Quality Control In Parkinson's Disease Models Via Mitochondrial UPR

**DOI:** 10.1002/advs.202408503

**Published:** 2025-07-20

**Authors:** Shuoting Zhou, Xi Xiong, Jialong Hou, Qi Duan, Yi Zheng, Tao Jiang, Jiani Huang, Haijun He, Jiaxue Xu, Keke Chen, Wenwen Wang, Jinlai Cai, Jingjing Qian, Huijun Chen, Weihong Song, XinShi Wang, Chenglong Xie

**Affiliations:** ^1^ Department of Neurology The First Affiliated Hospital of Wenzhou Medical University Wenzhou 325000 China; ^2^ Oujiang Laboratory Wenzhou Zhejiang 325000 China; ^3^ Key Laboratory of Alzheimer's Disease of Zhejiang Province, Institute of Aging Wenzhou Medical University Wenzhou Zhejiang China; ^4^ The Center of Traditional Chinese Medicine The Second Affiliated Hospital and Yuying Children's Hospital of Wenzhou Medical University Wenzhou 325027 China; ^5^ Department of Geriatrics, Geriatric Medical Center The First Affiliated Hospital of Wenzhou Medical University Wenzhou Zhejiang 325000 China

**Keywords:** mitochondria quality control, mitochondrial unfolded protein response, NAD^+^‐boosters, nicotinamide mononucleotide, Parkinson's disease

## Abstract

Serving as a pivotal hub for cellular metabolism and intracellular signaling, the mitochondrion has emerged as a crucial organelle whose dysfunction is linked to many human diseases, including neurodegenerative disorders, particularly Parkinson's disease (PD). However, whether mitochondrial quality control (MQC) can be targeted for therapeutic interventions remains uncertain. This study uses clinical samples, molecular biology techniques, pharmacological interventions, and genetic approaches to investigate the significance of NAD+ levels in cross‐species models of PD. These results reveal that treatment of rotenone‐incubated cells with NAD+ boosters (such as NMN, siCD38, and NAT) increases UPR^mt^/mitophagy‐related MQC, reduces pro‐inflammatory cytokine expression, inhibits apoptosis, and strengthen redox reactions. In vivo, NMN supplementation inhibits motor deficit and forestalls the neuropathological phenotypes of MPTP‐induced PD mice, which are required for the *atf4*‐related mitochondrial UPR pathway. Notably, bulk omics signatures and metabolomic profiling analyses of the striatum reveal NMN‐induced transcriptional changes in genes and proteins involved in mitochondrial homeostasis. Thus, these findings demonstrate that the accelerated pathology in PD models is probably mediated by impaired MQC and that bolstering cellular NAD+ levels alleviates mitochondrial proteotoxic stress and mitigate PD phenotypes.

## Graphical abstract

Treatment of MPTP‐incubated cells with NAD+‐boosters increase the UPR^mt^/mitophagy‐related mitochondria quality control (MQC). Disturbed plasma UPR^mt^‐mitophagy‐mediated MQC profiles in PD patient samples. NMN inhibits motor deficit and forestalls neuropathology phenotypes of PD mice, which is required the *atf4*‐medicated UPR^mt^ pathway. Bulk omics signatures and metabolomic analysis of the striatum reveale NMN involves in mitochondrial homeostasis characteristics.

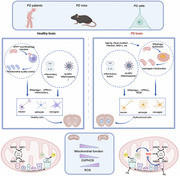

## 1 Introduction

Parkinson's disease (PD), pathologically characterized by the loss of dopaminergic (DA) neurons in the substantia nigra pars compacta and the presence of protein aggregates termed Lewy bodies, is the second most prevalent progressive multisystem neurodegenerative disorder.^[^ ^1^, ^2^ ^]^ Mitochondrial dysfunction is a crucial factor in PD pathogenesis and is associated with various neurodegenerative diseases.^[^ ^2^ ^]^ The initial evidence for mitochondrial dysfunction in PD was reported in the 1980s by Schapira et al., who identified defects in complex I within the cells and tissues of patients with PD.^[^ ^3^ ^]^ Maintaining mitochondrial fitness and homeostasis is essential for the survival and function of organisms. Eukaryotic cells have developed sophisticated mitochondrial quality control (MQC) mechanisms to identify, repair, and eliminate abnormal or misfolded proteins and dysfunctional mitochondria.^[^ ^4^ ^]^ Failure of these MQC pathways has been implicated in the pathogenesis of PD and other neurodegenerative conditions.^[^ ^5^ ^]^ If the repair is out of order, misfolded proteins accumulate in the brain, leading to their oligomerization into toxic multimeric complexes and contributing to the onset of PD.^[^ ^6^ ^]^ Despite a mounting understanding of the molecular mechanisms of mitochondrial dysfunction and quality control, many critical aspects of PD remain inadequately understood.

Nicotinamide adenine dinucleotide (oxidized form, NAD^+^) is present in all known life forms and serves as an indispensable cofactor for redox reactions involved in cellular bioenergetics, genomic stability, mitochondrial homeostasis, adaptive stress responses, cell survival, and so on.^[^ ^7^ ^]^ Mitochondria isolated from animal and postmortem human brain tissue show age‐dependent functional decline, increased oxidative damage, and disrupted membrane potential.^[^ ^8^ ^]^ NAD^+^ plays a crucial role in mitochondrial homeostasis, which involves mitochondrial biogenesis, mitochondrial unfolded protein response (UPR^mt^), mitophagy, and nuclear‐mitochondrial communication.^[^ ^9^ ^]^ Decreased NAD^+^ concentration plays a key role in brain aging and neurodegenerative disorders; hence, NAD^+^ replenishment improves mitochondrial function and reduces the accumulation of damaged mitochondria in premature aging models.^[^ ^10^ ^]^ Thus, as described in the previous sections, NAD^+^ is essential for the linking of mitochondrial biogenesis and quality control, thereby sustaining mitochondrial homeostasis in the brain.

Multiple protective mechanisms have evolved to identify and combat mitochondrial dysfunction and maintain the health of organisms.^[^ ^11^ ^]^ Among these, UPR^mt^ is essential for protecting the mitochondria from stress injury by inducing a dedicated transcriptional response in the nucleus to restore proteostasis.^[^ ^12^ ^]^ The maintenance of mitochondrial protein homeostasis is pivotal to mitochondrial fitness. Upon proteotoxic stress, the mitochondria elicit UPR^mt^, a nuclear transcriptional response that activates mitochondrial chaperones to reestablish homeostasis in the mitochondria.^[^ ^13^ ^]^ In detail, the translation initiation factor eIF2α is phosphorylated by one of four eIF2α‐specific kinases, such as GCN2. This phenomenon promotes the activation of transcription factors ATF4, CHOP, and ATF5. Although the true relationship between these transcription factors during mitochondrial stress remains to be determined, all three are indispensable for the induction of genes associated with the UPR^mt^ pathway.^[^ ^14^ ^]^ Impairments in mitophagy, the process of selective mitochondrial degradation by autophagy that contributes to the gradual accumulation of damaged mitochondria, have also been reported in PD.^[^ ^15^ ^]^ To keep mitochondrial networks and functions intact, cells have evolved quality control systems that eliminate superfluous mitochondria via the UPR^mt^ and mitophagy pathways.^[^ ^16^ ^]^

Neurons harboring the LRRK2 G2019S mutation in the PD model exhibit mitochondrial dysfunction and a reduction of approximately 30% in NAD^+^ levels.^[^ ^17^ ^]^ Meanwhile, DA neurons derived from induced pluripotent stem cells expressing GBA mutants display a decreased cytosolic NAD^+^/NADH ratio, likely due to a decrease in the NAD^+^ synthetic enzyme NMNAT2.^[^ ^18^ ^]^ Further evidence supporting that NAD^+^ deficiency is prevalent in many, if not all, instances of PD results from a study reporting that overactivation of the NAD^+^ Kinase SARM1 may lead to NAD^+^ depletion and axonal degeneration.^[^ ^19^ ^]^ In addition to the hyperactivation of SARM1, NAD^+^‐consuming PARP levels are upregulated in the cerebra and cerebrospinal fluid of patients with PD.^[^ ^20^ ^]^ All these results underscore the important role of NAD^+^ in PD pathogenesis and indicate that NAD^+^ augmentation may forestall PD‐related pathologies.^[^ ^18^, ^21^, ^22^ ^]^

Recent studies show NAD^+^ supplementation enhances SIRT3‐mediated mitigation of dopaminergic oxidative stress and mitophagy, while nanomaterial‐mediated mitochondrial nicotinamide mononucleotide delivery activates SIRT1/PGC‐1α to ameliorate MPP^+^‐induced mitochondrial dysfunction in PD models.^[^ ^23^, ^24^ ^]^ However, NMN‐related therapies still lack sufficient evidence and model application in PD. On the other hand, NAD^+^ strategies require further exploration in downstream UPR^mt^‐related MQC.^[^ ^21^ ^]^ Whether defects in MQC serve as disease drivers and whether boosting mitochondrial biogenesis through NAD^+^ supplementation offers neuroprotection in PD remain unclear. In addition, the discrepancy between individual NAD^+^ precursors (e.g., NMN, siCD38, and NAT) in PD models is not well understood and warrants further investigation.

## 2 Results

### 2.1 NAD^+^ Boosters are Beneficial for PD‐related Cell Models

**Figure 1:**
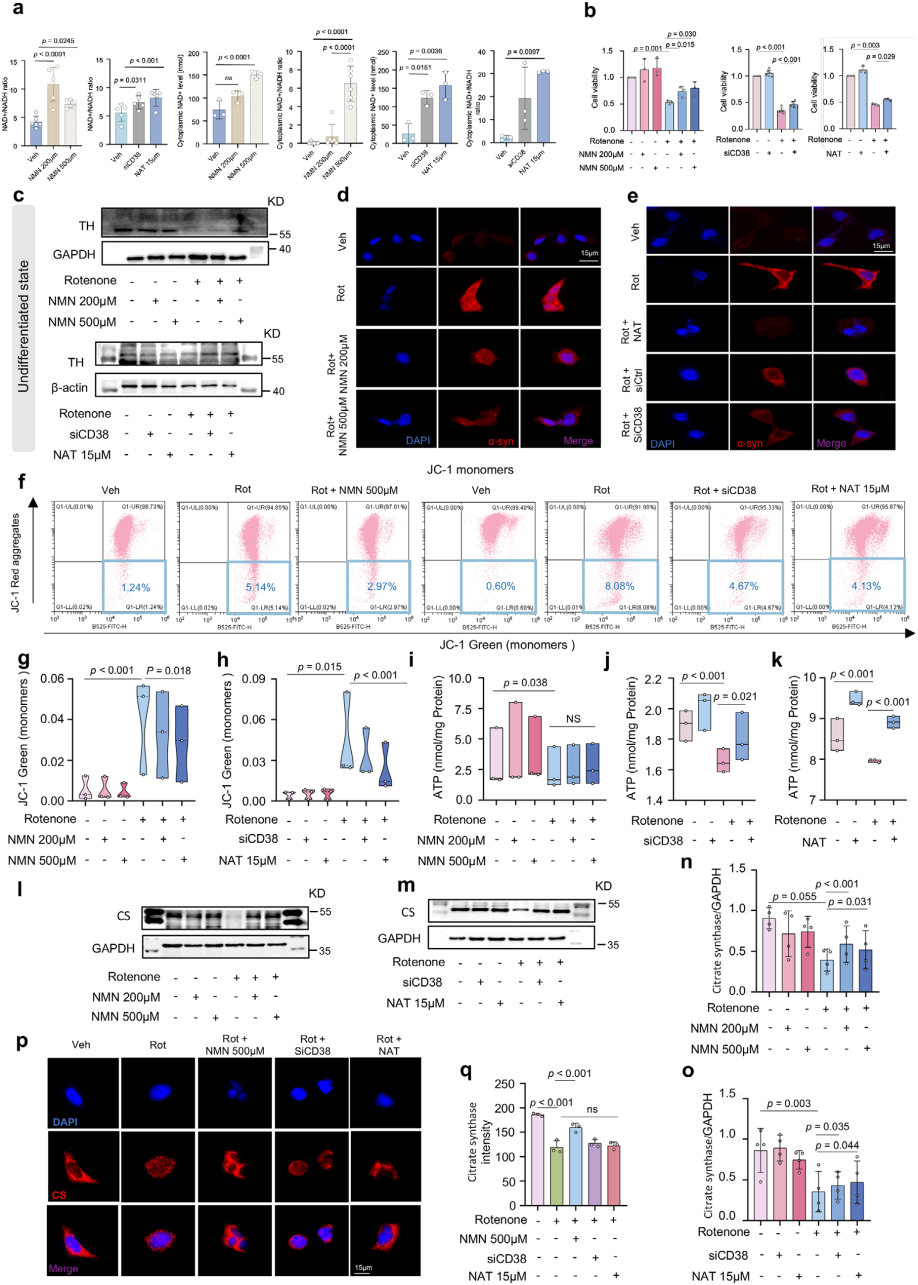
NAD+ boosters are beneficial for PD‐related cell models: a) Relative NAD^+^ and NADH levels in SH‐SY5Y cells (n = 3 independent samples; One‐way ANOVA). b) SH‐SY5Y cells’ survival rate was determined by CCK8 assay (n = 3; n = 6; n = 3; n = 6 independent samples; One‐way ANOVA). c) Western blot data showing changes of expression of TH when SH‐SY5Y cells were in an undifferentiated state (n = 3 independent samples; One‐way ANOVA). β‐actin and GAPDH was used as loading control for TH. d,e) Changes of α‐syn signals in different subgroups (n = 3 independent samples; One‐way ANOVA). For (d,e), scale bars, 15 µm. f) Flow cytometry analysis of mitochondrial membrane potential (ΔΨm) using JC‐1. The boxed percentage represents the proportion of cells with JC‐1 monomers in the total population. g,h) Quantification of changed percentages of green JC‐1 monomers in each group of cells, which is an indicator of low membrane potential. (n = 3 independent samples; One‐way ANOVA). i–k) Changes in intracellular ATP levels in each group (n = 3 independent samples; One‐way ANOVA). l,m) Western blot data showing changes of expression of Citrate synthase (n = 3 independent samples; One‐way ANOVA). n,o) Quantification of changes in Citrate synthase for each group of cells (n = 3 independent samples; One‐way ANOVA). GAPDH was used as loading control for CS. p) Changes of Citrate synthase signals in different subgroups. For (p), scale bars, 15 µm. q) Quantification of changes in Citrate synthase for each group of cells (n = 3 independent samples; One‐way ANOVA). Data are shown as mean ± SEM. The *p* values are indicated on the graphs. NS, not significant.

Following the observation of diminished NAD^+^ levels in PD models,^[^ ^18^ ^]^ we investigated whether this decline triggers accelerated neurodegenerative phenotypes in PD. We increased cellular NAD^+^ levels several times by incubating SH‐SY5Y cells with NAD^+^ boosters, including NMN, siCD38, and NAT, a chemical class of NAMPT activators (**Figures** **1**a and S1a, Supporting Information), and subsequently assessed intra‐ and extracellular NAD^+^ concentrations using an ELISA kit. This is in line with several studies on disease conditions,^[^ ^25^, ^26^ ^]^ suggesting that NMN, siCD38, and NAT are highly sensitive to NAD^+^ supplementation. Furthermore, we isolated the cytosolic and mitochondrial fractions to measure NAD^+^ and NADH levels. In the cytosol, all treatments significantly increased NAD^+^ levels and NAD^+^/NADH ratio. Notably, cytosolic NADH levels decreased in the siCD38 and NAT groups but increased in both NMN‐treated groups (Figure 1a and Figure S1a, Supporting Information). However, attempts to measure NAD^+^ and NADH levels in the mitochondria have been unsuccessful, likely because of their low abundance. This remains an intriguing area for future investigation. Moreover, the survival of rotenone/6‐OHDA‐treated cells decreased relative to that of the vehicle‐treated cells, and cell viability substantially changed after treatment with NMN (500 µM), siCD38, and NAT (15 µM) (Figure 1b and Figure S1b, Supporting Information). We examined the neuroprotective effects of these NAD^+^ boosters on rotenone‐induced PD cells. Tyrosine hydroxylase (TH) immunoblots revealed that the DA neuron index in the rotenone‐treated cells remarkably decreased. Treatment with NMN, siCD38, and NAT relieved PD‐related pathologies, such as increased TH levels both in undifferentiated and differentiated states (Figure 1c and Figure S1c–f, Supporting Information). In addition, the immunostaining data showed a higher a‐synuclein intensity in the rotenone‐treated cells than in the vehicle‐treated cells. These anomalies were reversed by approximately 50% after treatment with NMN, siCD38, and NAT (Figure 1d,e and Figure S1g,h, Supporting Information), indicating that NAD^+^ boosting can relieve PD‐related cellular pathologies. Since mitochondrial dysfunction has long been considered a key contributing factor in the pathogenesis of PD, we hypothesized that NAD^+^ boosters could mitigate impaired mitochondria and promote their homeostasis in PD cells. Here, we used JC‐1 dye to assess mitochondrial membrane potential (MMP, Δ*Ψ*_m_), green fluorescence (monomeric JC‐1) indicates low Δ*Ψ*m, while red fluorescence (JC‐1 aggregates) reflects high Δ*Ψ*m.^[^ ^27^ ^]^ Rotenone treatment increased the green fluorescence of JC‐1, indicating lower MMP, which consistent with the previous results.^[^ ^28^, ^29^ ^]^ Then, treatment with NMN, siCD38, and NAT partially abolished this ratio (Figure 1f–h and Figure S1i, Supporting Information), suggesting that NAD^+^ boosters prevent the collapse of MMP and maintain mitochondrial fitness. Moreover, the electron transport chain produced high levels of adenosine triphosphate (ATP) after treatment with NMN, siCD38, and NAT (Figure 1i–k). One prevailing strategy for estimating mitochondrial content is to measure the levels of citrate synthase (CS), a common marker of mitochondrial capacity, to normalize a given bioenergetic readout. As shown in Figure 1l, the representative blot shows a dramatic reduction in CS after treatment with 200 and 500 µM NMN, but the difference did not reach statistical significance. Notably, the activity of the CS protein was lower in the rotenone‐treated cells than in the vehicle‐treated cells, but it significantly increased after treatment with NMN, siCD38, and NAT (Figure 1l–o), indicating that the mitochondrial content was enhanced in the context of NAD^+^ boosters’ augmentation. Analogously, immunofluorescence analysis showed mild to moderate increased intensity of CS after treatment with 500 µM NMN, whereas treatment with siCD38 and NAT did not affect CS after rotenone treatment (Figure 1p,q). The differences in the effects of NMN, siCD38, and NAT on CS fluorescence intensity may be due to the off‐target effects of small‐molecule compounds. Off‐target effects occur when molecular compounds bind to targets other than the intended pharmacological targets, which may lead to unpredictable effects.^[^ ^30^ ^]^ It also showed that NMN had a stronger effect than siCD38 and NAT.

### 2.2 Disturbed Plasma UPR^mt^‐Mitophagy Pathways in Patients with Idiopathic PD

Studies over the past decade have verified that mitochondrial homeostasis is vital for neuroprotection in patients with PD disease. However, only a few studies have reported the circulating levels of UPR^mt^‐mitophagy‐related MQC pathways in patients with PD.^[^ ^31^, ^32^ ^]^ Under physiological or disease conditions, UPR^mt^‐mitophagy processes are the primary MQC pathways that handle stress resistance, as both are responsive to similar forms of mitochondrial stress.^[^ ^33^ ^]^ Our group previously found that patients with PD exhibit higher plasma levels of PINK1 and Parkin than healthy controls (HC).^[^ ^31^ ^]^ However, integrated studies examining both the UPR^mt^ and mitophagy targets are lacking. A total of 154 participants including 78 patients with PD (66.5 ± 10.5 years of age, 52.6% male) and 76 normal controls (63.9 ± 8.42 years of age, 41.3% male) were enrolled in this study (Table S1, Supporting Information). The basic information collected included age; sex ratio; height; weight; BMI; education; disease duration; UPDRS, MMSE, HAMA, HAMD, RBDQ‐HK, and ADL scores; and UPR^mt^‐mitophagy pathway protein levels. Notably, no significant differences in age, sex ratio, height, or education were found between the PD and HC groups, indicating that the patients in each group were comparable. Details of the basic characteristics of these participants are provided in Table S1, Supporting Information.

**Figure 2:**
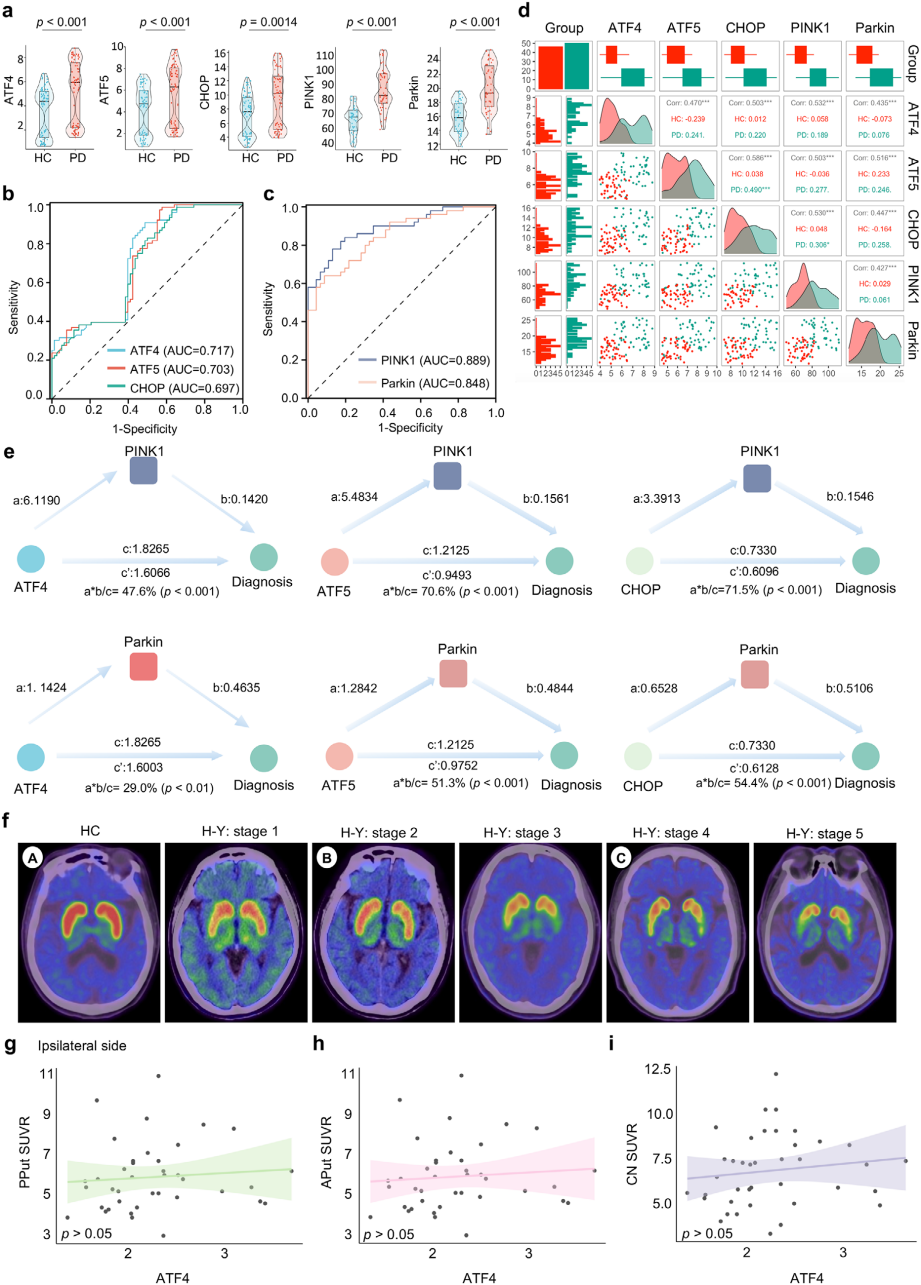
Disturbed plasma UPR^mt^‐mitophagy pathways in idiopathic PD subjects: a) Plasma levels of UPR^mt^ (ATF4, ATF5, CHOP) and mitophagy (PINK1, Parkin) biomarkers in patients with PD and matched healthy controls (HC) in cohorts a and b, respectively. Cohort a (HC = 76, PD = 78); cohort b (HC = 46, PD = 50). b) Corresponding receiver operating characteristic (ROC) curves for the UPR^mt^ biomarkers ATF4, ATF5, and CHOP. c) Corresponding ROC curves for the PD patients and healthy control's mitophagy (PINK1, Parkin) biomarkers. d) Multi‐group correlation analysis of the UPR^mt^ biomarkers in cohort b with the mitophagy biomarkers. e) Mediation analysis demonstrating the relationship between the UPR^mt^, the mitophagy, and the AD diagnosis. f) Representative ^18^F‐FP‐CIT DAT‐PET images of ipsilateral and contralateral sides of the PD and HC participants in different brain regions in Cohort c. Caudate nucleus, CN; Anterior putamen, AP; Posterior putamen, PP; Hoehn‐Yahr: H‐Y. g–i) Associations between of plasma ATF4 levels with ^18^F‐FP‐CIT DAT‐PET SUVR value in the PP (g), AP (h), and CN (i) regions of PD participants. *P* values and *R* coefficients are derived from Spearman correlations. Data are shown as mean ± SEM. The *P* values are indicated on the graphs. NS, not significant.

Moreover, patients with PD had significantly higher plasma concentrations of ATF4, ATF5, CHOP, PINK1, and Parkin than the controls (**Figure** **2**a and Table S2, Supporting Information). We next examined whether the plasma levels of individual UPR^mt^ proteins and mitophagy profiles could be distinguished between patients with PD and controls (Figure 2b,c and Table S3, Supporting Information). For the UPR^mt^ proteins, we found an area under the ROC curve (AUC) of 0.717 (95% CI: 0.635–0.799) for ATF4, 0.703 (95% CI: 0.620–0.786) for ATF5, and 0.697 for CHOP (95% CI: 0.614–0.780; Figure 2b). Similarly, regarding mitophagy markers, ROC tests compared the PD group with the HC group (Figure 2c), and the AUC was 0.889 (95% CI: 0.825–0.953) for PINK1 and 0.845 (95% CI: 0.773–0.923) for Parkin, indicating that the UPR^mt^‐mitophagy pathway is a suitable diagnostic marker panel. Moreover, after adjusting for age, sex, and disease duration, multivariate linear regression analysis revealed positive correlations of ATF4, ATF5, and CHOP indices with the levels of PINK1 and Parkin in all participants (Figure 2d).

Both UPR^mt^ and mitophagy are specific repair pathways within the mitochondria, with UPR^mt^ frequently acting as an upstream regulator of mitophagy.^[^ ^34^ ^]^ Subsequently, we investigated the role of mitophagy proteins in mediating the relationship between UPR^mt^ and PD diagnosis. The results demonstrated that the relationship between ATF4 and PD was mediated by PINK1 and Parkin, with an approximate proportion of mediation of 47.6% (*p* < 0.001) and 29.0% (*p* < 0.001), respectively (Figure 2e). Consistent with these results, our findings suggested that the association between ATF5/CHOP and PD diagnosis was also partially mediated by PINK1 and Parkin (Figure 2e). Notably, a multitude of PET radiotracers was used to assess nigrostriatal presynaptic dopamine transporter (DAT) integrity, which has been shown to correlate with the density of DA neurons. ^18^F‐FP‐CIT PET is a sensitive marker that can assess the striatal DAT to detect degenerative parkinsonism.^[^ ^35^ ^]^ Visual and semiquantitative analyses showed that the ^18^F‐FP‐CIT‐PET standardized uptake value ratios (SUVR) were lower in the posterior putamen (PP), anterior putamen (AP), and caudate nucleus (CN) of the PD group than in those of the HC group (Figure 2f and Table S4, Supporting Information), indicating that the uptake of this radiotracer was lower in the PD group than in the controls. Nevertheless, no significant correlations were found between ^18^F‐CIT‐PET SUVR in the PPut, APut, and CN regions and peripheral plasma ATF4, ATF5, CHOP, PINK1, and Parkin expression (Figure 2g–i, Figure S2, and Table S5, Supporting Information).

### 2.3 NAD^+^ Boosters Reinforce UPR^mt^/Mitophagy‐related MQC in PD Cells

**Figure 3:**
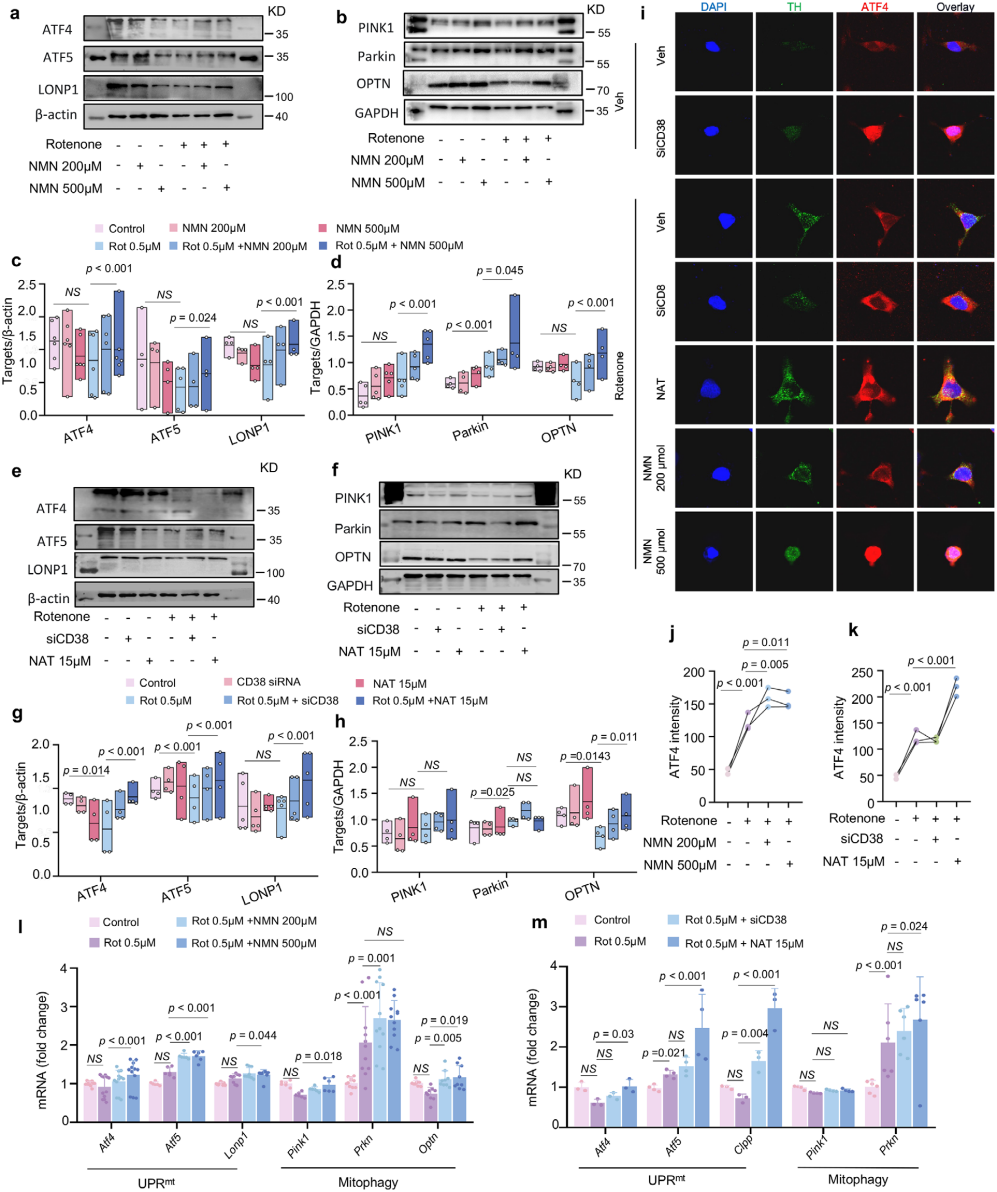
NAD^+^ boosters reinforce UPR^mt^/mitophagy‐related mitochondrial quality control in PD cells: a,b) Western blot data showing expression of mitophagy and UPR^mt^‐related proteins in SH‐SY5Y cells under different doses of NMN. c,d) Quantification of changes in the proteins shown in a, b (n = 3 independent samples; One‐way ANOVA). β‐actin was used as loading control for ATF4, ATF5 and LONP1. GAPDH was used as loading control for PINK1, OPTN and Parkin. e,f) Western blot data showing mitophagy and UPR^mt^‐related protein expressions in SH‐SY5Y cells under different elevated NAD^+^ pathways. g,h) Quantification of changes in the proteins shown in e, f (n = 4 independent samples; One‐way ANOVA). β‐actin was used as loading control for ATF4, ATF5 and LONP1. GAPDH was used as loading control for PINK1, OPTN and Parkin. i–k) Expression level of intracellular ATF4 under different treatments. For (**i**), scale bars, 15 µm (n = 3 independent samples; One‐way ANOVA). l,m) Real‐time PCR showing transcript levels of intracellular UPR^mt^ and mitophagy indicators under different treatments (n = 3 independent samples; One‐way ANOVA). Data are shown as mean ± SEM. The *p* values are indicated on the graphs. NS, not significant.

One of the core contradictions in PD may stem from mitochondrial dysfunction, which can be assessed through a series of mitochondrial stresses associated with reduced intracellular NAD^+^ levels.^[^ ^7^, ^36^ ^]^ NAD+‐boosting compounds may also induce the UPR^mt^‐mitophagy‐related MQC in various mammalian tissues.^[^ ^37^ ^]^ Thus, based on previous observations of disrupted plasma UPR^mt^‐mitophagy pathways in patients with PD, we further examined the impact of the generally identified NAD^+^ stimulators, such as NMN, siCD38, and NAT, on conserved MQC mechanisms involving UPR^mt^ and mitophagy. Our results showed that MQC was prominently ameliorated in the NMN‐treated PD‐related cells, as shown by the upregulation of UPR^mt^ proteins, such as ATF4, ATF5, and LONP1 (**Figure** **3**a,c), as well as mitophagy proteins (e.g., PINK1, Parkin, and OPTN) (Figure 3b,d), presumably to maintain protein homeostasis within the mitochondria. We also hypothesized that siCD38 and NAT would improve MQC in PD cells by activating UPR^mt^ and mitophagy proteins in a manner similar to that of NMN treatment. Expectedly, we observed a marked increase in UPR^mt^ and mitophagy proteins in SH‐SY5Y‐rotenone‐induced cells after siCD38 and NAT treatment (Figure 3e–h). Notably, the expression of some targets, such as PINK1 and Parkin, remained unchanged, although they showed a tendency. Similarly, immunostaining for ATF4 puncta revealed an elevated ATF4 signal in the SH‐SY5Y cells treated with NMN, siCD38, and NAT (Figure 3i–k). Meanwhile, ATF4 expression in the nucleus was significantly higher after treatment with siCD38, 15 µmol NAT, and 500 µM NMN than after treatment with rotenone alone, indicating ATF4 traffic to the nucleus to activate the UPR^mt^ response under these treatments. Next, we broadened the analysis of mRNA latitude and found an increase in several UPR^mt^ and mitophagy transcripts mediated by NMN, siCD38, and NAT (Figure 3l,m); however, no effect on the mitophagy transcript of *pink1* was observed. Results of western blot, immunostaining, and qPCR data indicated that NAD^+^ boosters enhanced MQC by upregulating the UPR^mt^ and mitophagy pathways in PD cell models.

### 2.4 Restoration of NAD^+^ Decreases Apoptosis and Enhances Oxidation Resistance

**Figure 4:**
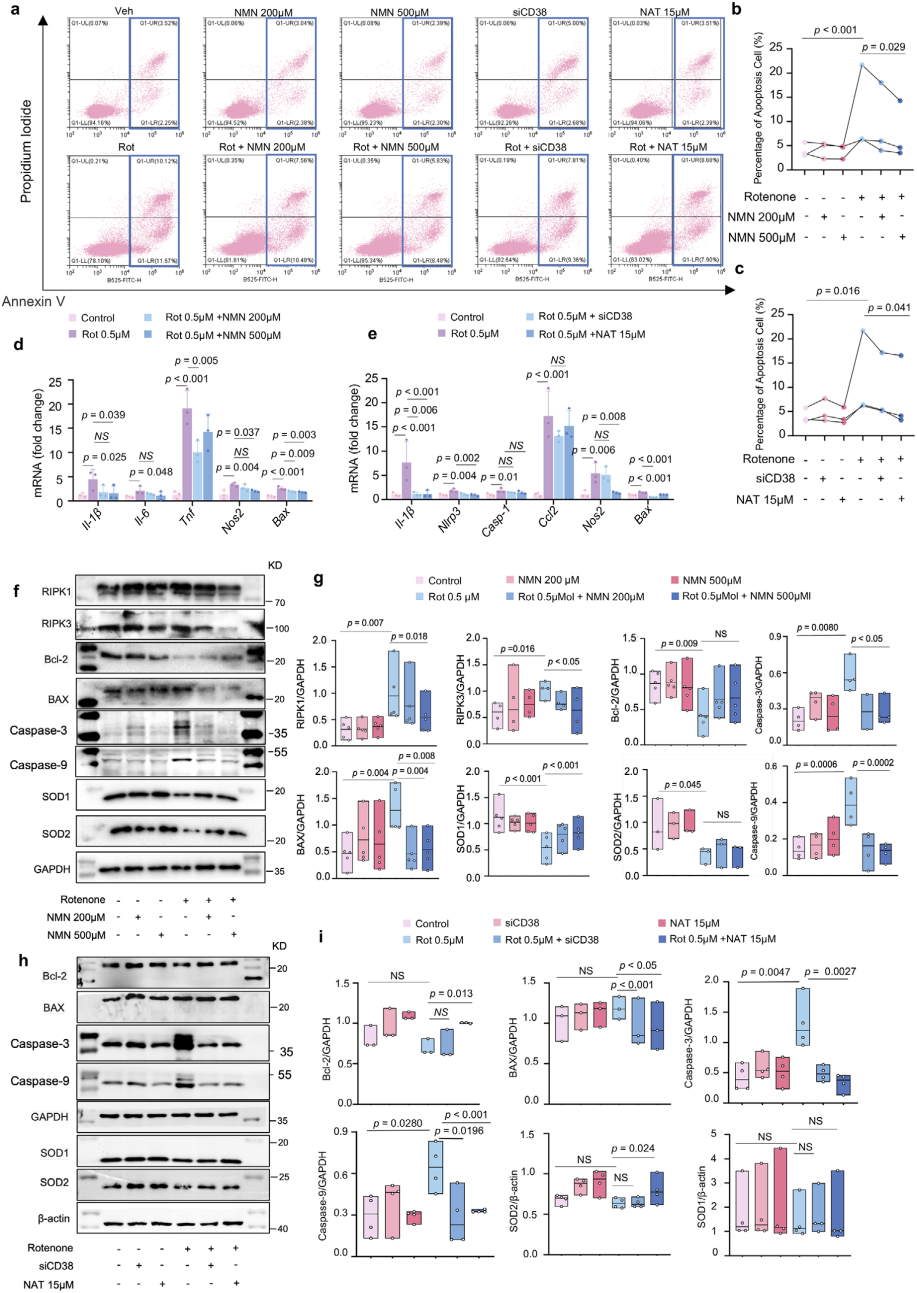
Restoration of NAD^+^ increases redox reactions and decreases apoptosis: a–c) Apoptosis in different groups of cells by detecting Annexin V/PI in flow cytometry (n = 3 independent samples; One‐way ANOVA). d, e) Real‐time PCR showing transcript levels of intracellular inflammatory and apoptosis indicators under different treatments (n = 3 independent samples; One‐way ANOVA). f–i) Western blot data showing changes of expression of inflammatory‐ and apoptosis‐related proteins under NMN supplemention and si‐CD31 or NAT treatment (n = 3 independent samples; One‐way ANOVA). GAPDH was used as loading control for RIPK1, RIPK3, Bcl‐2, Caspase‐3, BAX, SOD1, SOD2 and Caspase‐9 in (g). β‐actin was used as loading control for SOD1and SOD2 in (i). GAPDH was used as loading control for Bcl‐2, Caspase‐3, BAX, and Caspase‐9 in (i). Data are shown as mean ± SEM. The *P* values are indicated on the graphs. NS, not significant.

Impaired mitochondria play a central role in necrosis, ferroptosis, and apoptosis. Several new findings targeting MQC offer unexplored opportunities to regulate cell death.^[^ ^38^ ^]^ In the present study, flow cytometry results showed that apoptosis increased under rotenone conditions. We also observed a slight decrease in cell apoptosis after treatment with NMN, siCD38, and NAT (**Figure** **4**a–c), indicating that NAD^+^ boosters may attenuate apoptosis in PD cells and enhance cell viability. To further investigate the effects of NAD^+^ boosters on neuroinflammation, we performed qPCR to quantify neuroinflammation markers. NMN, siCD38, and NAT incubation decreased the levels of *Il‐1β*, *Tnf*, *Nos2*, *Bax*, and *Nlrp3* (Figure 4d,e). Subsequently, the effects of NMN on necrosis, apoptosis, and antioxidant protein levels were investigated. NMN treatment reduced necrosis marker (RIPK1 and RIPK3) and caspase‐3/9 levels, increased the Bcl‐2/Bax ratio, and elevated SOD1 concentrations rather than SOD2 protein, with no statistical difference (Figure 4f,g). Consistent with the NMN findings, a reduction in caspase‐3/9 and an increase in the Bcl‐2/Bax ratio and SOD2 concentration, all of which are significant antioxidant factors, were observed in the PD cells treated with siCD38 and NAT (Figure 4h,i). However, some factors such as SOD1 showed non‐statistically significant trends. Taken together, our results suggest that NAD^+^ boosters effectively inhibit apoptosis and enhance antioxidant pathways.

### 2.5 NAD^+^ Booster NMN Supplementation Inhibits Motor Deficit and Forestalls Neuropathology Phenotypes in MPTP‐induced Mice

**Figure 5:**
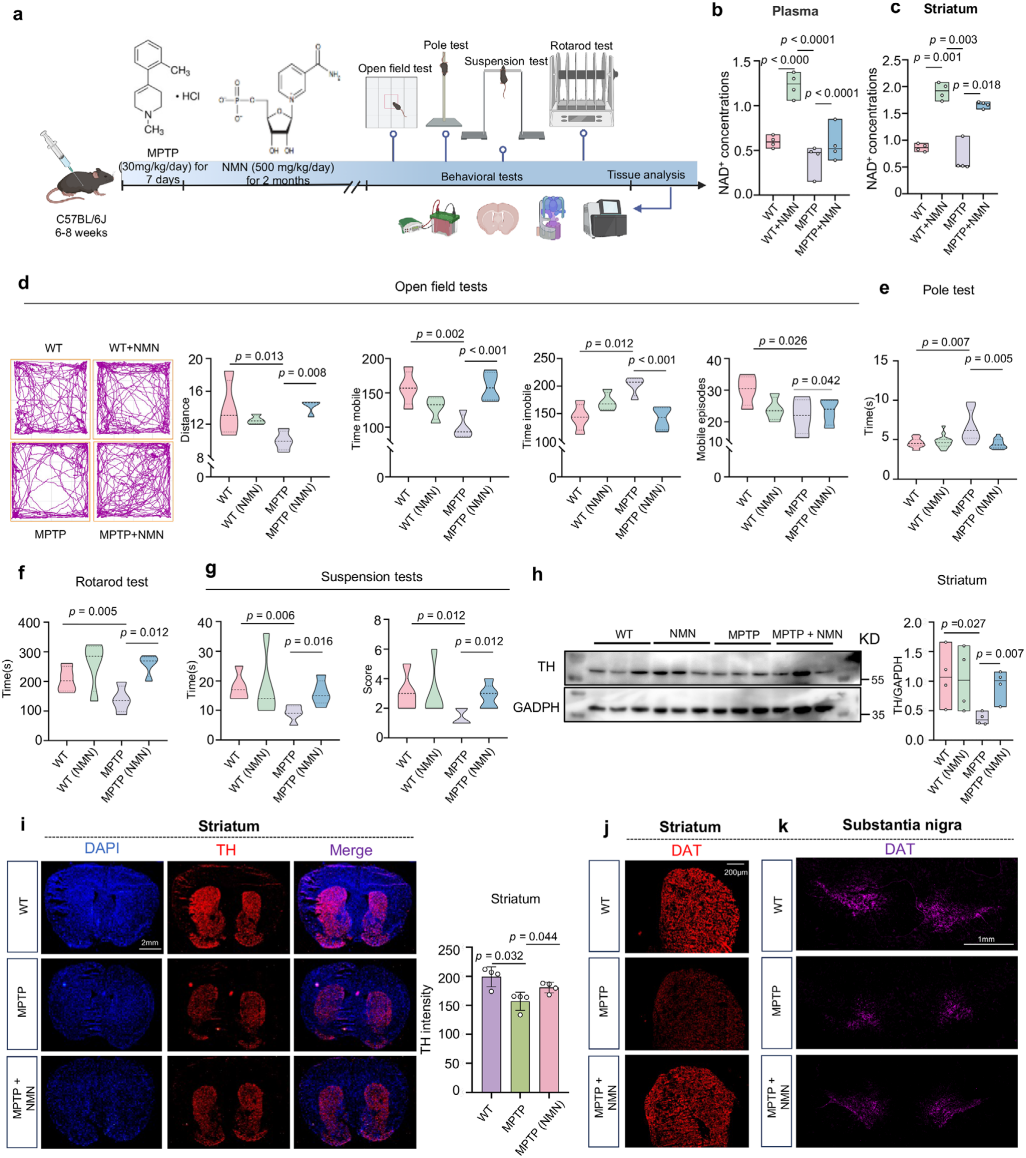
NAD^+^ booster NMN supplementation inhibits motor deficit and forestalls neuropathology phenotypes in MPTP‐induced mice: a) Flowchart of NMN intraperitoneal injection for treating MPTP‐induced mice model of PD. b) Plasma levels of NAD^+^ in different groups of mice after NMN treatment (n = 4 independent samples; One‐way ANOVA). c) Striatum levels of NAD^+^ in different groups of mice after NMN treatment (n = 4 independent samples; One‐way ANOVA). d–g) Bar plots of performance in the behavioral tests, including the open field test, pole test, rotarod test, and Suspension tests. (n = 4 independent samples; One‐way ANOVA). h,i) Levels of TH in the striatum of mice under NMN treatment by Western blot and IFC. For (h), scale bars, 2 mm (n = 4 independent samples; One‐way ANOVA). GAPDH was used as loading control for TH. j,k) Dopamine transporter levels in mice striatum and substantia nigra (n = 4). Data are shown as mean ± SEM. The *P* values are indicated on the graphs.

The neuroprotective role of NMN in PD has not been comprehensively elucidated in vivo. Given that the level of NAD^+^ was increased by NMN in the cell data, its role in protecting the PD cell model was more prominent than that of siCD38 and NAT. Hence, we evaluated the effects of NMN in an established MPTP‐induced PD mouse model of PD (**Figure** **5**a). As shown in Figure 5a, we administered MPTP (30 mg kg^−1^ day^−1^) to 2‐month‐old mice for 7 days, followed by NMN (500 mg kg^−1^ day^−1^) for 2 months. Tissues were collected for NAD^+^ measurement after NMN administration. The NAD^+^ levels in the plasma and striatum were significantly higher in the MPTP‐treated mice than in the vehicle‐treated mice (Figure 5b,c). Treatment with NMN improved the performance of the MPTP‐treated mice in open field tests (OFTs; Figure 5d), such as total distance traveled, time mobile, and mobile episodes, whereas the NMN‐treated mice showed less immobility time. In addition, the MPTP‐treated mice displayed mild motor impairment in the pole test, which is a standard paradigm used to assess motor deficiencies, and NMN significantly ameliorated the turning time and the total time (Figure 5e). The rotarod test is one of the oldest tests that has been extensively used to assess motor ability and coordination in mice since the 1950s.^[^ ^39^ ^]^ The MPTP‐induced mice exhibited a significantly lower latency to fall off the accelerating rotarod than the MPTP + NMN‐treated mice in all trials (Figure 5f). Suspension tests were performed using the MPTP‐induced mouse model to investigate the potential effect of NMN on antidepressant efficacy in animals. The results revealed a significant disparity in both the percentage of time spent immobile and the scores between NMN‐treated‐ and MPTP‐induced mice (Figure 5g). Together, these behavioral tests demonstrate that NMN significantly positively affects motor impairment and emotional performance in mice with PD.

Moreover, regarding the neuropathological phenotypes, the MPTP‐treated mouse model exhibited a substantial depletion of DA neurons, as visualized by TH immunoblotting, when compared with the control animals. Simultaneous abdominal injections of NMN partially prevented the loss of TH (Figure 5h). NMN also prevented the reduction of DA projections into the striatum in the MPTP model, as measured by the striatal optical density of TH fluorescence (Figure 5i). Next, we quantified the DAT levels in the striatum and substantia nigra using immunofluorescence staining. The results showed that NMN increased DAT intensity, indicating that NMN ameliorated the neuropathological characteristics of the MPTP‐treated mice (Figure 5j,k and Figure S3a,b, Supporting Information).

### 2.6 Omics Signatures of the Striatum with NMN in MPTP‐Induced PD Mice

**Figure 6:**
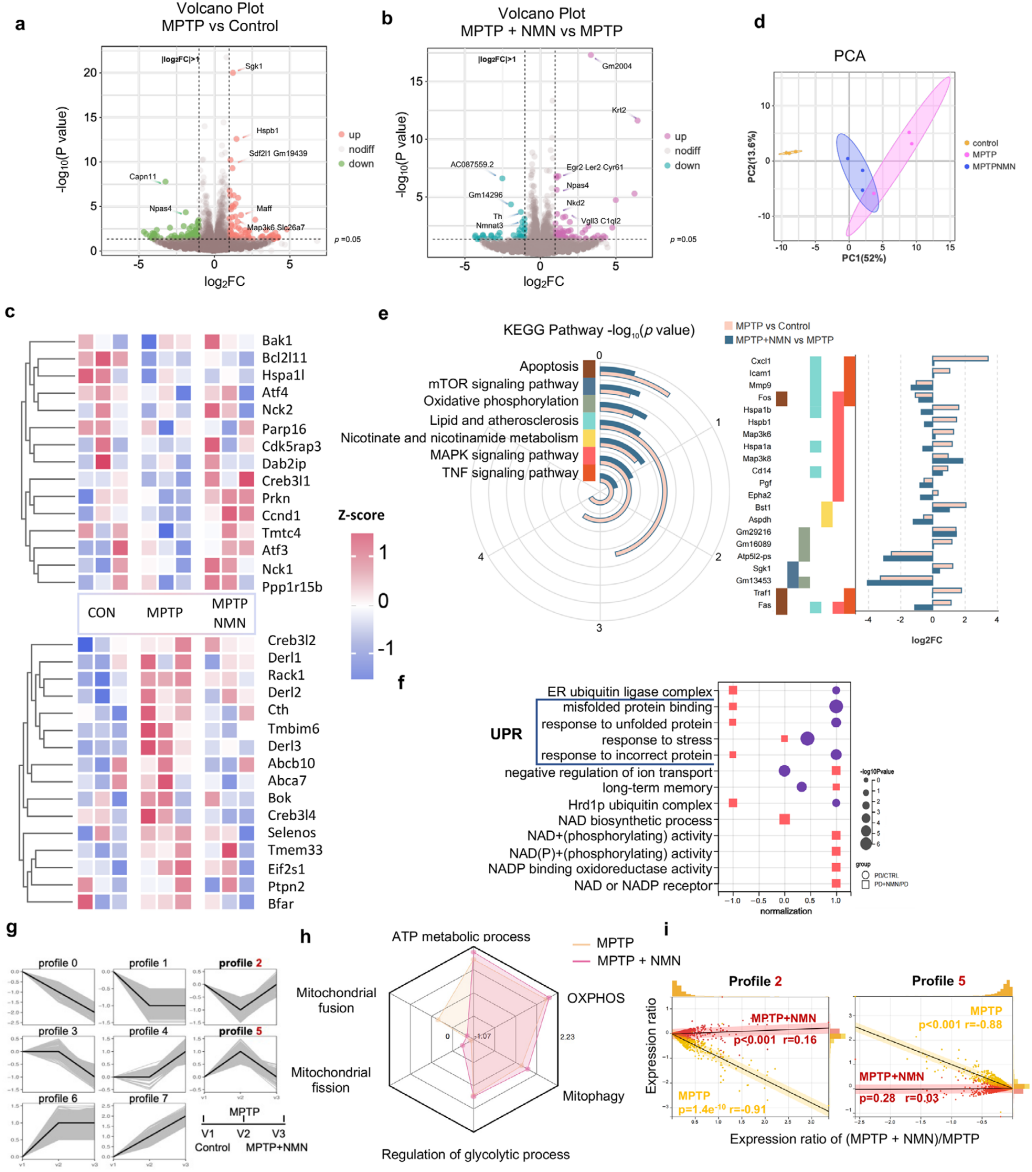
Omics signatures of the striatum with NMN in MPTP‐induced PD mice: a,b) differential genes for the MPTP and Control groups are shown in the volcano plots based on ‐log10 (*P* value) and log_2_FC, whereas MPTP+NMN and MPTP groups are displayed in b in the same way. c) Heatmap of differential genes in Control, MPTP, and MPTP+NMN groups. d) PCA analysis of Control, MPTP, and MPTP+NMN groups (n = 3). e) KEGG analysis of differential genes for the MPTP versus control group, and the MPTP + NMN versus MPTP group, and co‐enrichment of the pathways based on ‐log_10_(*P* value) and log_2_FC of related genes within the crucial KEGG pathways are shown in bar graphs. f) GO enrichment analysis of differential genes in the MPTP versus Control group, and MPTP + NMN versus MPTP group, and the core pathways are shown based on up‐ and down‐regulated normalized values and ‐log_10_(*P* value). g–i) Mfuzz trend analysis of Control, MPTP, and MPTP + NMN groups, and their expression trends were visualized in g, and all genes in different groups were plotted as scatter plots in i based on their MPTP + NMN/MPTP expression ratios and expression ratios with the control group for trends 2 and 5. h) The GSEA enrichment results of MPTP and MPTP + NMN groups according to mitochondria‐related pathways, and energy metabolism‐related pathways were visualized in radar plots. *P* values and *R* coefficients are derived from Spearman correlations. Data are shown as mean ± SEM. The *P* values are indicated on the graphs.

We further performed striatum bulk RNA‐sequencing analysis to identify the potential mechanisms through which NMN protects MPTP‐induced mice. The volcano plot depicts the upregulated and downregulated genes in WT vs. MPTP and MPTP vs. MPTP + NMN mice, respectively (**Figure** **6**a,b). The expression of these genes was altered by MPTP but reversed by NMN, as shown by the hierarchical clustering analysis (Figure 6c). Our findings revealed that NMN mitigated the MPTP‐induced disruption of the mitochondrial UPR, particularly involving genes such as *Atf4, Derl1, Ppp1r15b*, and *Nck1*. To confirm the reliability of the analysis, we performed principal component analysis and repeated the correlation evaluation, which revealed obvious differences in composition among the WT, MPTP, and MPTP + NMN groups (Figure 6d). To identify gene sets with similar biological activities that were altered in MPTP+NMN conditions, we performed KEGG and GO analyses of these genes to further illustrate their corresponding pathways and functions (Figure 6e,f). KEGG provides an enrichment analysis that considers all genes in a KEGG term using a non‐parametric, unsupervised method to estimate the variation in that gene set within each sample. The top differentially expressed gene (DEG)‐rich pathways induced by NMN included apoptosis, mTOR signaling, oxidative phosphorylation, lipid metabolism, MAPK signaling pathways, ER ubiquitin, misfolded protein binding, nicotinate metabolism, and TNF signaling pathways; DEG‐rich Gene Ontology (GO) terms, such as ER ubiquitin ligase complex, misfolded protein binding, response to unfolded protein, stress response, and response to incorrect protein, were also highly enriched in both comparisons (Figure 6e,f). To further examine the detailed genes involved in these pathways, we checked the number and *p*‐value of the identified DEGs. The log2‐fold changes and *p*‐values of 21 individual DEGs enriched in these pathways were also measured, including several UPR^mt^‐related heat shock protein (HSP) family members (Figure 6e, right panel). Through trend analysis, we observed that NMN reversed the expression of DEGs induced by MPTP in profiles 2 and 5, providing insights into the impact of NMN on gene expression in the different experimental groups (WT, MPTP, and MPTP + NMN) (Figure 6g). Combined data from profile 2/5 and gene set enrichment analysis (GSEA) revealed that transcripts encoding proteins involved in the ATP metabolic process, OXPHOS, regulation of glycolysis, and mitophagy were higher in the MPTP+NMN group than in the MPTP group (Figure 6h,i), all of which are implicated in mitochondrial homeostasis. Consequently, we provisionally deduced that NMN activated the UPR^mt^‐associated pathways in the MPTP‐induced PD mouse model. The UPR^mt^ response is primarily focused on reestablishing mitochondrial homeostasis. Although the UPR^mt ^pathway cannot be directly evaluated by GO or KEGG enrichment, we found that the cellular response pathways of UPR and essential UPR proteins were induced by NMN. Conversely, NMN enhances essential mitochondrial functions, including energy metabolism, thereby offering crucial energy support for MQC.

### 2.7 Mitochondria Homeostasis can be Improved by Increased NAD^+^ Biosynthesis in Mice

**Figure 7:**
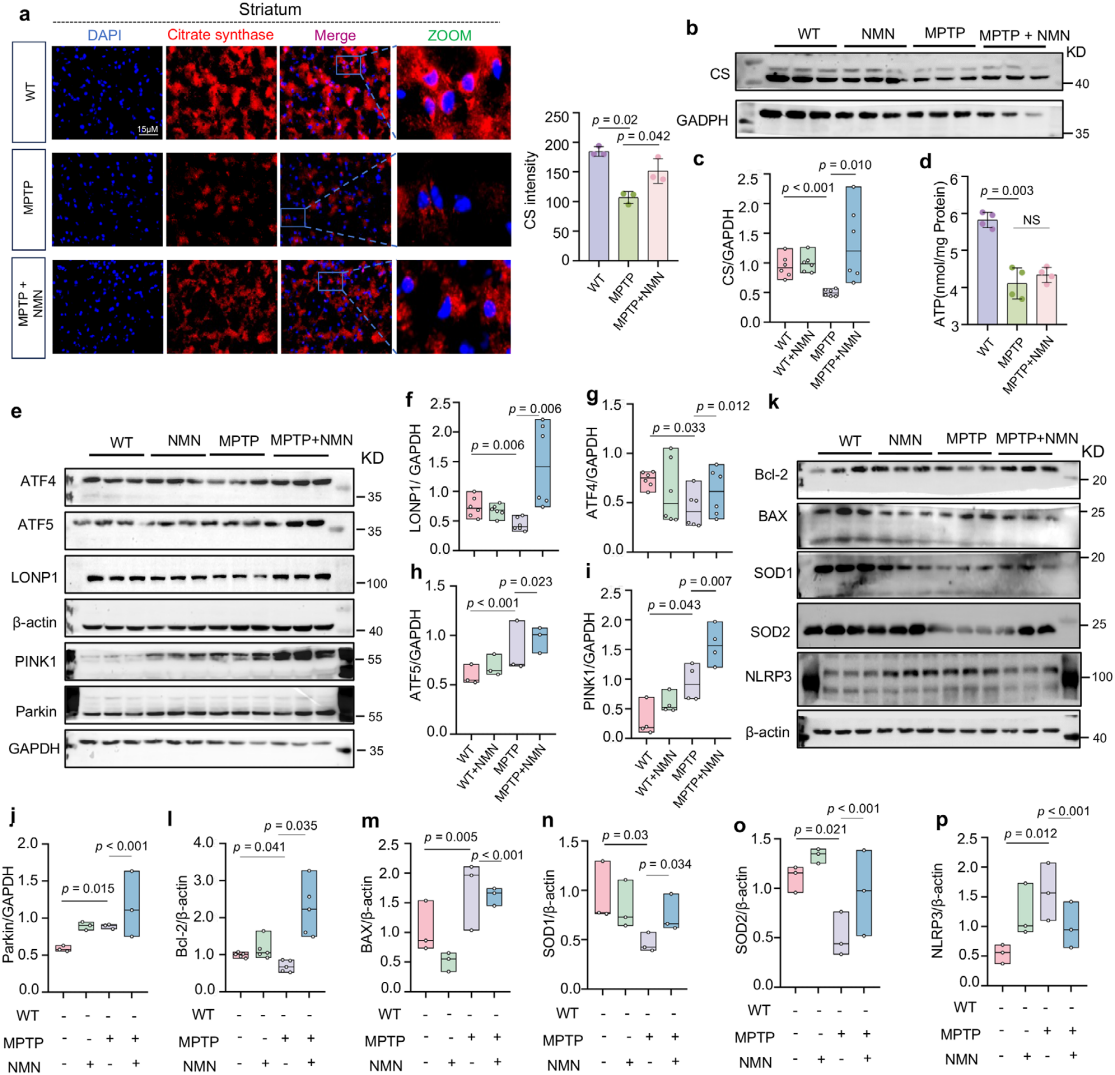
Mitochondrial quality control signature can be improved by increased NAD+ biosynthesis in mice: a) Immunofluorescence staining of Citrate synthase in the striatum of PD mice. For (c), scale bars, 15 µm (n = 3 independent samples; One‐way ANOVA). b,c) Effects of NMN on the expression level of Citrate synthase (n = 6 independent samples; One‐way ANOVA). d) Changes in intracellular ATP levels in each group (n = 4 independent samples; One‐way ANOVA). e–p) Effects of NMN on the expression levels of different proteins (n = 3 independent samples; One‐way ANOVA). β‐actin was used as loading control for ATF4, ATF5, LONP1, Bcl2, Bax, SOD1, SOD2 and NLRP3. GAPDH was used as loading control for PINK1 and Parkin. Data are shown as mean ± SEM. The *P* values are indicated on the graphs. NS, not significant.

Next, we attempted to gain insights into the molecular pathways underlying the therapeutic effects of NMN in mice. To address this, we explored its ability to influence mitochondrial homeostasis. Consistent with our previous report, the mice treated with MPTP showed a robust CS decline in the striatum by immunofluorescence and western blot analysis, which was significantly offset by NMN treatment (**Figure** **7**a–c). We showed that ATP was present in high amounts in the MPTP + NMN group compared with the MPTP‐vehicle group, indicating that NMN plays a crucial role in effective mitochondrial protection (Figure 7d). Next, we measured the UPR^mt^ and mitophagy pathways as the two major MQC tactics to evaluate whether their expression is also co‐regulated by NMN in the MPTP‐induced mouse model. The mice exposed to NMN had significantly higher levels of ATF4, ATF5, LONP1, PINK1, and Parkin than the mice treated with the vehicle (Figure 7e–j). Furthermore, immunoblotting revealed that NMN decreased the level of the neuroinflammatory marker NLRP3 and increased the Bcl‐2/Bax ratio and SOD1/2 concentrations (Figure 7k–p). In brief, MQC induction under NMN administration was observed at the protein level in the MPTP‐treated mouse model.

### 2.8 MPTP‐induced Reactive Gliosis and Synaptic Disruption were Attenuated by NMN Treatment

**Figure 8:**
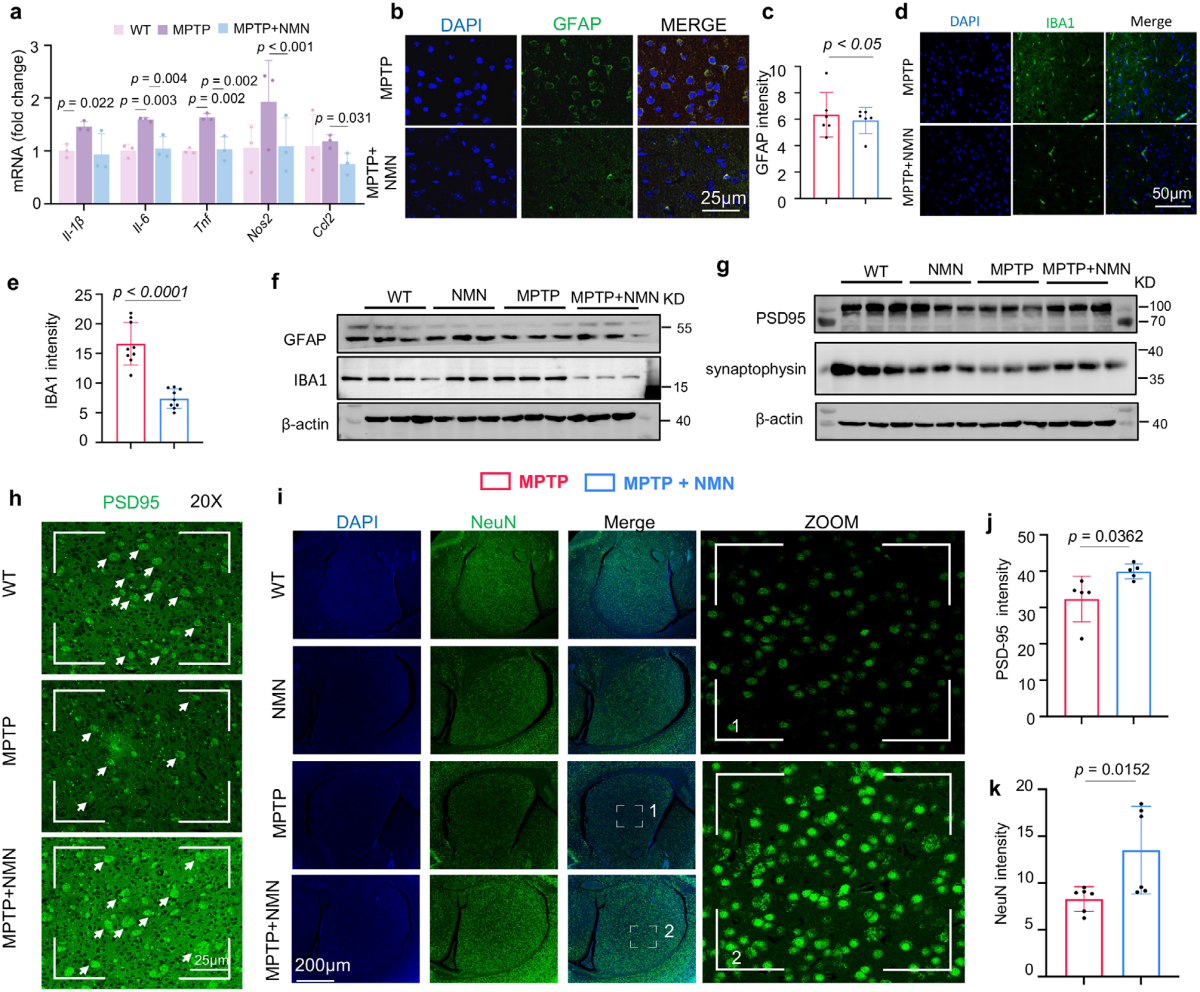
MPTP‐induced reactive gliosis and synaptic disruption were attenuated by NMN treatment: a) RT‐qPCR analysis revealed that the expression of genes such as *Il‐1β*, *II‐6*, *Tnf*, *Nos2*, *Ccl2*, and *Ccl2* (n = 3 independent samples; One‐way ANOVA). b–f) Effects of NMN on the expression level of IBA1 and GFAP (n = 3 independent samples; One‐way ANOVA). β‐actin was used as loading control. g,h,j) Effects of NMN on the expression level of synaptophysin and PSD95 (n = 5 independent samples; One‐way ANOVA). β‐actin was used as loading control. i,k) Effects of NMN on the expression level of NeuN (n = 6 independent samples; One‐way ANOVA). Data are shown as mean ± s.e.m. The *P* values are indicated on the graphs. NS, not significant.

Chronic neuroinflammation is a hallmark of PD pathophysiology. Postmortem inspection of patients with PD and experimental animal studies indicate that the activation of glial cells and increased pro‐inflammatory factor concentrations are common features of the PD brain.^[^ ^40^ ^]^ To evaluate the effect of NMN administration on MPTP‐induced neuroinflammation and glial activation, we analyzed the expression of pro‐inflammatory and glia‐associated genes after MPTP injection through RT‐qPCR, western blot, and immunohistochemistry. RT‐qPCR analysis revealed that the expression of *Il‐1β*, *II‐6*, *Tnf*, *Nos2*, *Ccl2*, and *Ccl2* considerably increased in the MPTP‐injected mice but significantly decreased in the NMN‐treated mice (**Figure** **8**a). Immunohistochemical analysis revealed that the immunoreactivity of GFAP and IBA1 decreased in the striatum after NMN treatment (Figure 8b–e). We performed immunofluorescence staining for the microglia‐specific markers Tmem119 and found that NMN inhibited the expression of Tmem119 (Figure S3c,d, Supporting Information). Similarly, immunoblotting revealed that NMN decreased the concentrations of the gliosis markers GFAP and IBA1 (Figure 8f and Figure S3e, Supporting Information). These data indicated that NMN supplementation considerably suppressed MPTP‐induced neuroinflammation and gliosis. In recent years, numerous causative genes and risk factors for PD have been identified and suggest that the loss of synaptic plasticity contributes to neurodegeneration and is a key player in PD.^[^ ^41^ ^]^ Biochemical analysis by fractionation of synaptosomes from MPTP + NMN mouse striata demonstrated a significant increase in the levels of synaptophysin and PSD‐95 (Figure 8g and Figure S3f, Supporting Information), pre‐ and post‐synaptic markers, respectively, indicating synaptic disruption retention with NMN administration. Consistent with these results, immunohistochemistry for NeuN and MAP2 revealed that the intensity of normal axons and dendrites decreased after MPTP injection, and this decrease was partially reversed by NMN pre‐administration (Figure 8h–k and Figure S3g,h, Supporting Information). Taken together, these results suggested that the administration of NMN attenuated not only MPTP‐induced neuroinflammation but also subsequent MPTP‐induced neurodegeneration.

### 2.9 NMN Attenuates PD‐Related Phenotypes through the ATF4 Pathway

**Figure 9:**
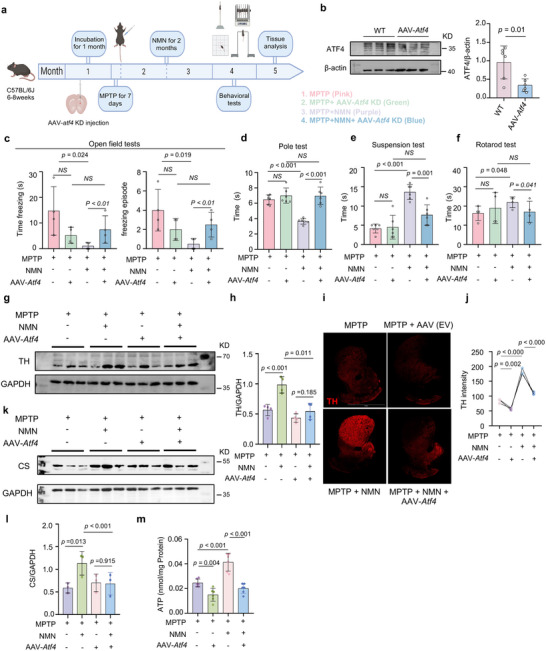
NMN attenuates PD‐related phenotypes through the ATF4 pathway: a) Experimental design to study the effect of *Atf4* knockdown in the striatum of NMN‐treated PD mice. b) ATF4 knockdown efficacy test in the striatum (n = 6 independent samples; One‐way ANOVA). β‐actin was used as loading control. c–f) Bar plots of performance in the behavioral tests, including the open field test, pole test, rotarod test and suspension tests (n = 4 independent samples; One‐way ANOVA). g–j) TH levels in the mice striatum. For (i), scale bars, 2 mm (n = 3 independent samples; One‐way ANOVA). GAPDH was used as loading control. k,l) Effects of NMN and *Atf4* knockdown on the expression level of CS in the striatum (n = 4 independent samples; One‐way ANOVA). GAPDH was used as loading control. m) Changes in intracellular ATP levels in each group (n = 6 independent samples; One‐way ANOVA). Data are shown as mean ± SEM. The *P* values are indicated on the graphs. NS, not significant.

As described above, NMN increases MQC and reinforces motor capacity in MPTP‐treated mice; however, the underlying mechanism remains unclear. Notably, NMN treatment significantly increased UPR^mt^ signaling at the protein level in both mammals and cells. Therefore, we speculated that NMN may attenuate PD‐related phenotypes via the ATF4‐UPR^mt^ pathway (**Figure** **9**a). We tested this hypothesis by injecting mice with adeno‐associated virus (AAV) to decrease ATF4 protein levels. Immunoblot analysis showed that 1 month after exposure to the virus, the striatum infected with AAV‐*atf4*‐KD expressed approximately 0.5‐fold lower amounts of ATF4 protein than the control tissue (Figure 9b and Figure S3i,j, Supporting Information), along with similar data in vitro (Figure S3k,l, Supporting Information). We then assessed the motor behavior of the MPTP‐induced mice after they were injected with *atf4‐*AAV‐KD. NMN improved the performance of the MPTP‐treated mice in the OPT, including freezing and freezing episodes; this benefit was abolished by *atf4* knockdown (Figure 9c). We found that the freezing time of mice in the OPT changed after AVV‐*atf4*‐KD, suggesting that *atf4* knockdown alone is involved in physiological emotion regulation. Next, we checked whether *atf4* knockdown affects motor capacity using the pole and rotarod tests. Similarly, as shown in Figure 9d,e, the movement disorder in the MPTP‐treated mice was mitigated by NMN treatment, and these phenotypes were abrogated by *atf4* knockdown. During the test period, knockout of *atf4* resulted in a significantly decreased time spent in the pole and rotarod, even with NMN administration, suggesting that NMN attenuates MPTP‐mediated motor loss through the ATF4 pathway. In addition, NMN significantly positively affected the emotional performance of the MPTP‐induced PD mice in the suspension test, which was also reversed by *atf4* knockdown. Our results showed that *atf4* knockdown alone did not lead to significant differences in the locomotor ability of the mice and had almost no effect on the locomotor function scores of the MPTP‐treated mice. Next, we investigated the effects of *atf4* deletion on striatal TH expression. TH immunoblotting and staining revealed lower TH levels in the brains of the MPTP + NMN + AAV‐*atf4*‐KD mice than in those of the MPTP + NMN control mice (Figure 9g–j). Next, we examined the CS levels by western blot. In contrast to the level of CS in the NMN‐treated group, which was higher than that in the MPTP‐treated group, a significantly lower CS level was observed in the *atf4* deficiency background (Figure 9k,l). Finally, the reduced ATP levels were ameliorated by NMN treatment, which was also required for *atf4* expression (Figure 9m). These findings suggest that the NAD^+^‐ATF4 axis protects mitochondrial homeostasis and preserves motor performance in mouse models of PD.

### 2.10 Treatment with NMN Remodels Metabolic Pathways of the Striatum in MPTP‐Induced PD Mice

**Figure 10:**
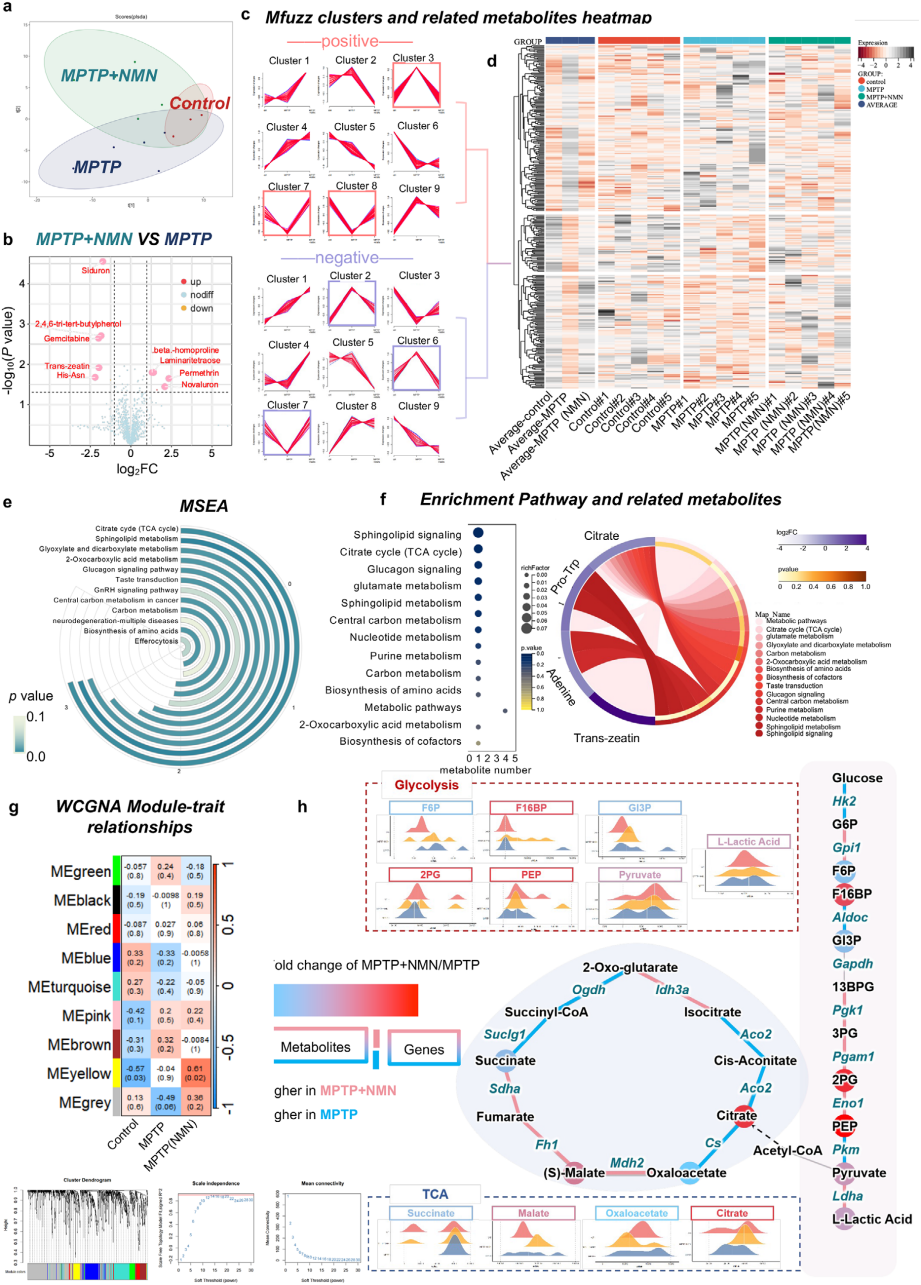
Treatment with NMN remodels metabolic pathways of the striatum in MPTP‐induced PD mice: a) OPLS‐DA analysis of Control, MPTP, and MPTP + NMN groups (n = 3). b) Metabolites for MPTP + NMN and MPTP groups based on ‐log_10_(*P* value) and log_2_FC are presented in the volcano plots. c,d) Mfuzz trend analysis of metabolites obtained from positive and negative ion patterns for different groups is presented in (c), and integration and visualization of critical metabolite subclusters' expression levels are plotted in the heatmap (d). e) MSEA enrichment analysis of differential metabolites in the MPTP + NMN vs MPTP group, and the pathway with the highest enrichment level was selected and visualized based on its p‐value. f) GO pathway enrichment analysis of differential metabolites in the MPTP + NMN vs MPTP group, and metabolites involved in multiple pathways were plotted as chordal plots based on their pathway interactions. g) WCGNA of control, MPTP, and MPTP + NMN groups, where the cluster dendrogram represents the metabolite groups identified by WGCNA. Meanwhile, the heatmap represents the correlation of different metabolite modules with NMN and MPTP. The critical metabolites in the Yellow, Grey, and Green modules are further analyzed in supple Figure 4. h) Combined metabolomics and transcriptomics analysis of Control, MPTP, and MPTP + NMN groups, where dots represent metabolite abundance, lines represent transcriptional expression of the enzymes, and the color differences indicate the degree of their high expression level in the MPTP or NMN groups. Then, peak plots indicate the respective degree of expression of all detected metabolites.

To further explore the protective mechanism of NMN treatment in the MPTP‐induced PD model, we performed a non‐targeted metabolomic analysis of the striatum across the different treatment groups. As shown in **Figure** **10**a, the components of the metabolites significantly changed after 8 weeks of NMN treatment compared with the MPTP group, based on the OPLSDA score plot with partial overlap in the positive electrospray ionization mode (ESI +). Next, the volcano map illustrates the overall trend of the differences in metabolites between the MPTP and MPTP+NMN groups (Figure 10b). To investigate how NMN rescued MPTP‐induced metabolite changes, we performed an Mfuzz trend analysis of the metabolites obtained in the ESI+ and ESI‐ modes. MFuzz is a type of soft clustering in which a sample can be assigned to multiple clusters with a probability cutoff. We selected the trend clusters whose expression levels were reversed by NMN compared with the MPTP group and merged them (Figure 10c and Figure S4a, Supporting Information), including clusters 3, 7, and 8 in the ESI+ as well as clusters 2, 6, and 7 in the ESI‐, respectively. The heatmap illustrates the variation in the expression of the selected metabolites, which increased or decreased in the hierarchical clustering analysis among the three groups, as determined using the MFuzz method (Figure 10d). The metabolic pathway and MSEA analysis for functional annotation and enrichment analysis of the selected metabolites showed that the most pertinent pathways in the MPTP and MPTP+NMN groups included the TCA cycle, sphingolipid metabolism, glyoxylate and dicarboxylate metabolism, 2‐oxocarboxylic acid metabolism, and glucagon signaling pathway, among others (Figure 10e). We then visualized the metabolites involved in regulating these pathways in a circos plot and found that citrate regulates multiple metabolic pathways, whereas prolyl, tryptophan, and adenine are involved in multiple sphingolipid pathways (Figure 10f). By combining the MSEA and pathway enrichment results, we preliminarily identified citrate as the key metabolite induced by NMN in the TCA cycle. Utilizing weighted gene co‐expression network analysis, we identified nine co‐expression modules, with the yellow and grey modules showing significant association with NMN administration (Figure 10g). Specifically, several proteins in the yellow and grey modules were upregulated considerably in the MPTP + NMN mice compared with the MPTP‐vehicle mice. These proteins included fructose 1,6‐bisphosphate, 2‐phosphoglycerate, phosphoenolpyruvate, malate, and citrate proteins. Conversely, fructose 6‐phosphate, glyceraldehyde‐3‐phosphate, succinate, and oxaloacetate were the top‐ranked hub metabolites that were decreased in the NMN group (Figure 10h and Figure S4b–d, Supporting Information). Functional annotation showed that the proteins in these modules were associated with cellular energy metabolic processes, such as glycolysis and the TCA cycle.

## 3 Discussion

Mounting evidence suggests that mitochondrial dysfunction plays a key role in PD pathogenesis.^[^ ^42^ ^]^ However, the underlying mechanisms remain unclear. This study indicates that patients with PD exhibit abnormalities in mitochondrial homeostatic proteins, primarily characterized by disrupted plasma UPR^mt^‐mitophagy pathways, including ATF4, ATF5, CHOP, PINK1, and Parkin. NAD^+^ supplementation (e.g., NMN, siCD38, and NAT) has been demonstrated to enhance MQC and alleviate inflammation‐related phenotypes. Mitochondrial homeostasis regulates numerous cellular functions, and alterations in disordered UPR^mt^‐mitophagy processes have been observed in PD models.^[^ ^43^, ^44^ ^]^ In the present study, we provide evidence for a direct interaction between NAD^+^ boosters and MQC in vitro and in vivo, demonstrating that NAD^+^ supplementation ameliorates PD‐related mitochondrial proteotoxicity, increases synaptic integrity in the striatum, and decreases glial activation and neurodegeneration in the brain. Furthermore, treatment with NMN improved MQC and cellular homeostasis in the brains of PD mice by activating UPR^mt^‐mitophagy signaling, suggesting that the neuroprotective effect of NMN may be mediated by the ATF4 pathway.

Remarkably, bulk omics features of the striatum with NMN in the MPTP‐treated mice showed that NMN induces transcriptional changes in genes and proteins involved in mitochondrial homeostasis, such as UPR^mt^‐related HSP family members, OXPHOS, mitophagy, and glycolysis regulation. Notably, the metabolomic data from these mice further confirmed alterations in mitochondrial metabolism after MPTP injection. NMN augmentation resulted in a notable enhancement of cellular energy metabolic processes, including glycolysis and the TCA cycle within the striatum. This strongly suggests enhanced mitochondrial protection in mice supplemented with NMN. In addition, UPR^mt^ itself does not directly involve the production or consumption of metabolites; thus, UPR^mt^ levels are more challenging to reflect through metabolomics than through transcriptomics. However, UPR^mt ^is closely associated with mitochondrial metabolic status. Here, our analytical approach to metabolomics aimed to reflect the indirect relationship between NMN and UPR^mt^ by assessing the restoration of mitochondrial functional homeostasis. Thus, based on our results, we speculate a highly favorable communication between NAD^+^ and UPR^mt^‐mediated MQC against MPTP‐induced toxicity. These findings suggest that enhancing mitochondrial function and proteostasis may reduce the detrimental proteotoxic stress in PD.

Increasing intracellular NAD^+^ concentrations has demonstrated protective effects against age‐related mitochondrial decline and associated diseases.^[^ ^9^, ^45^ ^]^ NAD^+^ serves as a coenzyme for various enzymes that regulate mitochondrial biogenesis, autophagy, and cellular stress responses.^[^ ^7^ ^]^ NAD^+^ consumption is driven by various pathological and physiological mechanisms, including oxidative stress and aging.^[^ ^46^ ^]^ We investigated the role of exogenous NAD^+^ supplementation in modulating MQC in PD models. Our findings indicate that NAD^+^ boosters such as NMN, siCD38, and NAT positively influence PD‐related cell models by enhancing UPR^mt^ and mitophagy pathways, improving redox reactions, and reducing apoptosis. The MPTP‐treated mice exhibited significant disturbances in the UPR^mt^ and mitophagy indices. Supplementation with the NAD^+^ booster NMN mitigated motor deficits, prevented neuropathological features, and alleviated glial activation and synaptic dysfunction in the striatum. Thus, perturbation of the UPR^mt^ and mitophagy proteins in the plasma of patients with PD may also account for the heightened mitochondrial proteotoxic stress observed in these PD models.

Here, we reported that patients with PD exhibited significantly elevated plasma concentrations of ATF4, ATF5, CHOP, PINK1, and Parkin compared with controls, indicating a potential perturbation of the UPR^mt^‐mitophagy pathways in PD. Furthermore, NAD^+^ boosters enhanced the activation of the UPR^mt^ and mitophagy pathways in the rotenone‐treated cell models and MPTP‐induced mice. Collectively, these findings suggest that the UPR^mt^‐mitophagy pathway plays a crucial role in maintaining mitochondrial homeostasis and may contribute to the progression of PD. Considering that the upregulation of an indicator in a given disease may be of dual natures, we believe that the upregulation of ATF4, ATF5, CHOP, PINK1, and Parkin in patients with PD may be a stress response triggered by mitochondrial dysfunction. Specifically, mitochondrial stress due to mitochondrial dysfunction in patients with PD activates the expression of the UPR^mt^ and mitophagy‐related genes through a negative feedback mechanism to enhance MQC and maintain cellular homeostasis. Hence, we suppose that the upregulation of these metrics is not the cause of mitochondrial dysfunction but is more likely to be a consequence of impaired mitochondria and tends to be protective in its function. One study reported that patients with Alzheimer's disease also display increased levels of serum and CSF PINK1 and serum BNIP3L‐related mitophagy biomarkers.^[^ ^47^ ^]^

A recent study on PD corroborated these findings, demonstrating that UPR^mt^ activation induced by mutant ornithine transcarbamylase or ATF5 overexpression confers protection against MPP^+^‐induced toxicity and ameliorates mitochondrial dysfunction.^[^ ^48^ ^]^ Increasing 4E‐BP1 expression or enhancing 4E‐BP1 activation protects neurons from misfolded protein stress and PD‐related toxicity by inducing the UPR^mt^.^[^ ^44^ ^]^ Using a *Caenorhabditis elegans* PD model, Cooper et al. found that *Pdr‐1* or *Pink1* mutations promote the survival of dopamine neurons by activating ATFS‐1‐dependent UPR^mt^, whereas ATFS‐1 disruption significantly diminishes neuron survival and accelerates the loss of dopamine neurons in this PD strain.^[^ ^49^ ^]^ In addition, overexpression of CLPP, a mitochondrial protease that participates in the UPR^mt^ pathway, regulates proteostasis in dopamine neurons of α‐Syn‐A53T transgenic mice and patients with PD, thereby reducing the mitochondrial oxidative damage and pathological accumulation of α‐Syn.^[^ ^50^ ^]^ These findings suggest that promoting the expression of UPR^mt^ may be an effective strategy for delaying the progression of PD. Regarding the mitophagy domain, studies over the last two decades have elucidated the complex interconnection between mitophagy and both sporadic and familial PD.^[^ ^51^, ^52^ ^]^ Several genetic studies have shown that mutations in key mitophagy regulators PINK1 and Parkin lead to early‐onset PD. The pharmacological boosting of mitophagy and promotion of the removal of damaged mitochondria are of interest for the development of disease‐modifying PD therapeutics.^[^ ^53^ ^]^ The current study supports the role of the UPR^mt^‐mitophagy pathways in regulating PD neuroprotection.

Activation of microglia and astrocytes in the PD brain leads to the release of inflammatory cytokines, promoting neurodegeneration.^[^ ^54^ ^]^ In the present study, our results indicated that MPTP‐induced glial activation and synaptic plasticity were attenuated by NMN treatment. Consistent with our findings, the synaptic disruption and anti‐neurodegenerative effects of NAD^+^ boosters have also been supported by other disease models. Treatment of APP/PS1 mice with NR for 5 months reduces the expression of pro‐inflammatory cytokines and improves synaptic plasticity, learning, and memory capacity.^[^ ^55^ ^]^ Supplementation with NMN ameliorates behavioral performance in fear conditioning and Morris water maze tests. Furthermore, NMN administration can attenuate apoptosis, inflammation, and oxidative responses in septic mice by regulating the SIRT1 pathway.^[^ ^56^ ^]^ NR increases neurogenesis, inhibits neurodegeneration, and restores hippocampal synaptic plasticity in 3xTgAD and 3xTgAD/Polβ^+/−^ mice.^[^ ^57^ ^]^ These results suggest that NAD^+^‐boosting ameliorates inflammation and improves synaptic plasticity. NMN and NR, two critical intermediates in the NAD^+^ biosynthesis pathway, have garnered significant attention in recent years.^[^ ^58^ ^]^ A previous study showed that NMN supplementation can significantly increase plasma NAD^+^ concentrations within 2.5 min. NMN can be directly transported across the cell membrane by Slc12a8 and then converted to NAD^+^ efficiently.^[^ ^59^ ^]^ Long‐term oral administration of NMN (up to 300 mg kg^−1^ for more than 1 year) has been demonstrated to be safe and well tolerated in normal mice.^[^ ^60^ ^]^ Recent studies have further corroborated these findings, indicating that a single oral dose of NMN does not cause any specific deleterious effects in healthy men.^[^ ^61^ ^]^ Based on these results, we selected NMN as a safe regimen to mitigate symptoms in PD mouse models. However, some limitations still exist and will need to be improved upon. First, several studies indeed have revealed the role of NAD^+^ in ameliorating PD and the association of NAD^+^ with UPR^mt^.^[^ ^62^, ^63^, ^64^ ^]^ To address this issue, we systematically elaborated the specific role of the NAD^+^‐ATF4 axis in the pathogenesis of PD, confirming that the ATF4‐mediated activation of the UPR^mt^ pathway is a key mechanism of the process, which was validated by multi‐latitude data both from clinical samples of PD patients and PD models. Second, this study only examined NMN in mice, future work should test other NAD^+^ precursors in PD models, such as NAT and CD38 deletion.

## 4 Conclusion

This study elucidated the mechanisms underlying neurotoxin‐induced PD and revealed that mitochondrial dysfunction is a critical element in the pathogenesis of the disease. Our findings demonstrate that NAD^+^ precursors ameliorate mitochondrial proteotoxic stress in PD. Among the available NAD^+^ precursors examined, NMN has emerged as a promising therapeutic candidate because of its high bioavailability and minimal toxicity.

## 5 Experimental Section

### Chemicals and Reagents

MPTP and NMN were purchased from Macklin; rotenone was purchased from Aladdin; NAT was purchased from MCE; cell counting kit‐8 (lipo2000 (GK20005) and MitoSOX (GC68230) were purchased from GLPBIO; siCD38 was purchased from GenePharma; BCA protein assay kit (P0010), ATP assay kit (S0026B), NAD^+^/NADH assay kit with WST‐8 (S0175), mitochondrial membrane potential assay kit with JC‐1 (C2006), DAPI staining solution (C1006), antifade mounting medium (P0128M), primary antibody diluent (P0023A), RIPA lysis buffer (P0013B), PMSF (ST506), QuickBlock blocking buffer for immunol staining (P0260), QuickBlock primary antibody dilution buffer for immunol staining (P0262), and QuickBlock secondary antibody dilution buffer for immunofluorescence (P0265) were purchased from Beyotime; Annexin V‐FITC/PI kit (FXP018Pro‐050) was purchased from 4A BIOTECH; RNA simple total RNA kit (DP419) was purchased from TIANGEN; qRT Master Mix (RTQ‐100) and qPCR Master Mix (QST‐100) were purchased from Toroivd; protein‐free rapid blocking buffer (PS108) was purchased from Epizyme; and paraformaldehyde (BL539A) and TRIS_EDTA antigen retrieval solution (50X) (BL617A) were purchased from Biosharp.

### Cell Cultures, NAD^+^ Booster Incubation, and Transfections

Undifferentiated SH‐SY5Y cells were cultured in high‐glucose/DMEM cell culture medium containing 10% fetal bovine serum and 1% penicillin–streptomycin solution and maintained in a cell incubator with 5% CO2 and 95% air at 37 °C.^[^ ^65^ ^]^ SH‐SY5Y cell differentiation was induced and maintained using 20 µM RA for 24–48 h in 1% FBS and 1% penicillin–streptomycin solution DMEM/F12 medium. NMN at 200 and 500 µM, NAT at 15 µM, and siRNA transfected with CD38 (forward, 5′‐CUCACAUGGUGUGGUGAAUTT‐3′ and reverse, 5′‐AUUCACCACACCAUGUGAGTT‐3′) were administered to increase intracellular NAD^+^ levels. The concentration of rotenone was 0.5 µM, and the concentration of 6‐OHDA was 500 µM. NMN, NAT, and siCD38 were added prior to rotenone treatment.

### Cell Viability Assay

This method was modified from a published protocol.^[^ ^66^ ^]^ Cells were seeded in 96‐well plates at a density of 6000 cells per well. After 2 days, the culture solution in the plates was completely absorbed, 100 µL of CCK8 reagent diluted at 1:10 was added to each well, and then the cells were incubated at 37 °C for 2–4 h. The optical density (OD) values were measured at 450 nm using an enzyme reader. Cell viability was calculated using the formula [(As ‐ Ab) / (Ac ‐ Ab)] * 100%. As: absorbance of the experimental well; Ac: absorbance of the control well; Ab: absorbance of the blank well.

### Flow Cytometry

Apoptosis was detected using an Annexin V‐FITC/PI Kit.^[^ ^67^ ^]^ In accordance with the manufacturer's instructions, the cells were digested with pancreatic enzymes, centrifuged at 1000 rpm for 5 min at 25 °C, and then washed with pre‐cooled PBS. Binding buffer resuspension cells were added, the concentration was adjusted to 1–5 × 10^6^ mL^−1^, and 100 µL was collected. Annexin V‐FITC 5 µL was added, mixed, and then incubated at room temperature without light for 5 min. Then, 10 µL of PI and 400 µL of PBS were added and mixed, followed by flow cytometry.

### Animal Experiments and NMN Treatments

Male C57BL/6 mice, 6–8 weeks old (weight, 25 ± 2 g), were purchased from Zhejiang Charles River Experimental Animal Technology Co. (Zhejiang, China). The mice were housed in a room temperature environment (22 ± 2 °C) under a 12‐h light/dark cycle (08:00, lights on; 20:00, lights off) with free access to water and food. MPTP (30 mg kg^−1^, ip.) was administered intraperitoneally once daily for 1 week to induce PD. NMN (500 mg kg^−1^, ip.) was administered once daily for 2 months. All animal care and experiments were approved by the Committee on the Ethics of Animal Experiments of the Wenzhou Medical University (WYYY‐AEC‐YS‐2023‐0004).

### Parkinson's Disease Behavior Test on Mice–Pole Test

The mice were placed on a pole (height, 50 cm; diameter, 1 cm) and pretrained three times to ensure that they descended smoothly when placed upside down. The time from the start of the trial to the bottom of the pole was recorded.^[^ ^68^ ^]^

### Parkinson's Disease Behavior Test on Mice–Open Field Test

This method was modified from a reported protocol.^[^ ^69^ ^]^ Prior to the experiment, the mice were placed in the behavioral laboratory for > 6 h for adaptation. In the open field experiment, the mice were gently placed in the center of an experimental box (50 cm × 50 cm). A camera was used to record the movements of the mice, and the experiment was performed for 15 min. Anymaze7.20 software was used to analyze the total distance moved, mobile time, immobile time, and mobile episodes in the experimental box.

### Parkinson's Disease Behavior Test on Mice–Suspension Test

The mouse forepaws were hung from a wire placed horizontally 30 cm above the floor. The time required to land was also recorded. The scores are 0 for 0–4 s, 1 for 5–9 s, 2 for 10–14 s, 3 for 15–19 s, 4 for 20–24 s, 5 for 25–29 s, and 6 for more than 30 s.^[^ ^70^ ^]^

### Parkinson's Disease Behavior Test on Mice–Rotarod Test

The mice were placed on a rotating bar in a stationary state, the speed was increased from 5 to 10 rpm, and the mice were habituated for 5 min for 3 days consecutively. For the formal test, the speed was increased from 5 to 40 rpm, and the test was performed three times at intervals of 10 min. The time at which the mouse fell off the bar was recorded.^[^ ^71^ ^]^

### Virus Information and Injections

AVV‐ATF4 was purchased from GeneChem. After the mice were anesthetized, their head skin was prepared. The head of each mouse was fixed using a special mouse brain stereotactor. The surgical area was disinfected three times with iodophor cotton balls. The incision was centered on the median line, approximately 1 cm long. The skin was cut, the subcutaneous tissue and muscle were bluntly separated, and the fontanel was exposed. Marked by the anterior bregmel, the injection coordinates of the striatum brain region were as follows:^[^ ^72^ ^]^ AP 0.5 mm, ML 2.2 mm, DV 2.6 mm. The striatum was located and drilled, 1 µL of virus was injected into the striatum at a rate of 0.2 µL min^−1^, and the retrograde needle was left in place for 5 min. The incision was closed after further disinfection with iodophor.

### Detection of NAD+ and NADH Levels

NAD^+^ levels in the cells and striatum were determined using the WST‐8 NAD^+^/NADH assay kit (Beyotime, S0175).^[^ ^73^ ^]^ An appropriate number of cells was first inoculated into a 6‐well plate. After 2 days of drug treatment, an appropriate amount of NAD^+^/NADH extract was added and incubated. The freshly harvested striata were immediately added to the extraction solution and ultrasonically disrupted. The samples were then centrifuged at 12 000 g for 5–10 min at 4 °C, and the supernatant was collected. A 100 µL aliquot of the supernatant was heated at 60 °C for 30 min, and 20 µL of the heated and unheated supernatant was added to a 96‐well plate. Then, 90 µL of the prepared alcohol dehydrogenase working solution was added and then incubated at 37 °C for 10 min. Each well was added with 10 µL of color developing solution and then incubated away from the light for 30 min. The OD value was detected at a wavelength of 450 nm using an Enzymoleter. Concentrations were calculated using a standard curve. The cytoplasmic fractions were isolated using the nuclear‐cytosol extraction kit (Applygen, P1200), followed by the steps described above.

### Mitochondrial Parameters Assay–ATP detection assay

Intracellular and striatal ATP levels were determined using an ATP assay kit.^[^ ^74^ ^]^ For the cells, an appropriate amount of lysate was added to the well plate and homogenized by sonication. An appropriate amount of mitochondria was added to the freshly harvested striatum and sonicated. The supernatant was then collected by centrifugation at 12 000 g for 5 min at 4 °C and transferred to a new tube. A 20 µL aliquot of the supernatant was mixed with 100 µL of the working dilution for the detection of ATP, and the brightness was measured using a chemiluminescence meter (luminometer). Concentrations were calculated from the standard curve.

### Mitochondrial Parameters Assay–Mitochondrial Membrane Potential Detection

The MMP assay kit with JC‐1 was used to detect MMP.^[^ ^75^ ^]^ Cells were resuspended in 0.5 mL of culture medium, mixed with 0.5 mL of JC‐1 staining solution, incubated at 37 °C for 20 min, and then centrifuged at 600 g for 4 min at 4 °C. The supernatant was discarded and then washed twice with JC‐1 staining buffer. Finally, an appropriate amount of JC‐1 staining buffer was added for resuspension and then detected by flow cytometry.

### Mitochondrial Parameters Assay–Mitosox

Adherent cells were cultured on a sterile cell crawler, and excess culture medium was aspirated. The dye working solution (100 µL) was added and gently shaken to completely cover the cells and incubated for 5–30 min. The dye‐working solution was aspirated and washed two to three times with culture medium for 5 min each. Observations were performed under a fluorescence microscope.

The brain slices were first antigen repaired. Then, 30 µL of the staining working solution was added dropwise to the brain slices, incubated for 30 min, and then washed three times with PBS for 5 min each time. Observations were conducted under a fluorescence microscope.

### Western Blot Analyses

For Western blot experiments,^[^ ^75^ ^]^ SH‐SY5Y cells were treated as previously outlined and then a mixture of RIPA lysate (Beyotime, P0013B), PMSF (Beyotime, ST506) and phosphatase inhibitors (Applygen, P‐1260) were prepared at a ratio of 100:1:1 and added to the cells. The cells were gently scraped and transferred to an EP tube for a 15‐min lysis period. Mouse striatum extracts were separated following standard protocols. The tissues were lysed using RIPA buffer, followed by centrifugation at 12 000 rpm for 30 min at 4 °C. The supernatants were collected for subsequent analysis. Protein concentration was determined using the BCA Protein Assay Kit (Beyotime, P0010). Based on the measured protein concentration, the protein solution was mixed with PBS and loading buffer, then boiled at 100 °C for 10 min. For SDS‐polyacrylamide gel electrophoresis (SDS‐PAGE) analysis (Miniprotean; Bio‐Rad), 30 µg of protein from each sample was loaded onto the gel and subjected to electrophoresis at 10V for 30 min, followed by 80V for 90 min. The proteins separated on the gel were then transferred to PVDF membrane (Millipore, Bedford, MA, USA). Protein‐free rapid blocking buffer (Epizyme, PS108) was used to block the membrane for 20 min at room temperature. Recommended concentrations of primary antibodies were incubated with the blocked membrane at 4 °C overnight. Membranes were washed and incubated with peroxidase‐conjugated antibodies for 1 h at room temperature. After the washing step, chemiluminescence was visualized using the e‐Blot chemiluminescence imaging system (Shanghai, China). The optical density of the strip was analysed using Image J software. Antibodies used were: anti‐TH (AB152, Sigma‐Aldrich), anti‐LC3B (L7543, Sigma‐Aldrich), anti‐Parkin (A0968, ABclonal), anti‐PINK1 (A7131, ABclonal), anti‐OPTN (A1845, ABclonal), anti‐SOD2 (A19576, ABclonal), anti‐ATF4 (DF6008, Affinity), anti‐ATF5 (DF3480, Affinity), anti‐LONP1 (DF12119, Affinity), anti‐RIPK1 (AF7877, Affinity), anti‐RIPK3 (DF10141, Affinity), anti‐Bcl‐2 (AF6139, Affinity), anti‐BAX (AF0120, Affinity), anti‐IL‐1β (AF5103, Affinity), anti‐Cleaved‐Caspase 1 (AF4022, Affinity), anti‐Caspase‐3 (AF6311, Affinity), anti‐Caspase‐9 (AF6348, Affinity), anti‐Citrate synthetase (DF13222, Affinity), anti‐IBA1 (DF6442, Affinity), anti‐GFAP (DF6040, Affinity), anti‐DAT Polyclonal (22524‐1‐AP, proteintech), anti‐SOD1 (#37385, Cell Signaling Technology), anti‐PSD95 (#3450, Cell Signaling Technology), anti‐synaptophysin (#5461, Cell Signaling Technology), anti‐NLRP3 (WL02635, Wanleibio), anti‐α‐syn (ab51253, Abcam), anti‐GAPDH (AP0063, Bioworld), anti‐β‐actin (AF0003, Beyotime) antibody, horseradish peroxidase (HRP)‐labeled goat anti‐rabbit IgG (A0208, Beyotime) and HRP‐labeled goat anti‐mouse IgG (A0216, Beyotime).

### Reverse Transcription‐Quantitative Polymerase Chain Reaction

Total RNA was extracted using the Total RNA Extraction Kit provided by TianGen, and for cells, 1 mL of RZ lysate was added to each cell dish, blown until clear, and then transferred to a centrifuge tube. For striatum, 1 mL of lysate was added per 50 – 100 mg of tissue, followed by sonication and crushing, and the supernatant was collected in a centrifuge tube. It was left to stand for 5 min at room temperature, 200 ul of chloroform was added to each tube, shaken vigorously for 15 s and left to stand for 3 min at room temperature. The tubes were then centrifuged at 4 °C, 12 000 rpm for 10 min, the top layer of liquid was removed and added to anhydrous ethanol, transferred to a centrifuge column and purified by adding RD and RW. The RT‐PCR reaction was then performed using the qRT Master Mix supplied by Toroivd. Quantitative polymerase chain reaction was performed using the qPCR Master Mix supplied by Toroivd. 95 °C for 10 s followed by 40 cycles (95 °C for 15 s and 60 °C for 30 s).

Following primers were used: Human ATF5: 5’‐ACTACCACCACCACCACTACCAC‐3’ and 5’‐GCCAGCAAGTCCAGAGTATCCAAG‐3’; Human ATF4: 5’‐AATACAACTGCCCTGTTCCCGATTC‐3’ and 5’‐GCCAGCAAGTCCAGAGTATCCAAG‐3’; Human LONP1: 5’‐ GCTGTTTGTGGAGACATCCCTGAG‐3’ and 5’‐AAGGCTCTGGCGAAGGTGTAGG‐3’; Human CLpP: 5’‐GTGGGATGTGACAGAGAGCAGTTTC‐3’ and 5’‐ATCTCAGGCGACAGGTCTCCATC‐3; Human PRKN: 5’‐GTCGCTTAGCAACCACCTCCTTG‐3’ and 5’‐GTCGCTTAGCAACCACCTCCTTG‐3’; Human PINK1: 5’‐AAGAGACCATCTGCCCGAGTAGC‐3’ and 5’‐GGAGCCAGCCAACCATCTTGTC‐3; Human OPTN: 5’‐GCGGCAGGTGTGGCTTTGATAG‐3’ and 5’‐GGTGAGGAAGTTGGGTTTACAGAGG‐3’; Human GAPDH: 5’‐TGCCCTCAACGACCACTTTG‐3’ and 5’‐CCTCTTGTGCTCTTGCTGGG‐3’; Human CD38: 5’‐TCACCGTGTTAGCCAGGATAGGG‐3’ and 5’‐TTCCATAGTTTCCACCGCCTTGC‐3’; Human IL‐1β: 5’‐TTCGACACATGGGATAACGAGG‐3’ and 5’‐TTTTTGCTGTGAGTCCCGGAG‐3’; Human NLRP3: 5’‐GCTTGCCGACGATGCCTTC‐3’ and 5’‐CTGTCATTGTCCTGGTGTCTTCC‐3’; Human CASP1: 5’‐GGTGCTGAACAAGGAAGAGATGG‐3’ and 5’‐CCTGAGGAGCTGCTGAGAGTC‐3’; Human IL‐6: 5’‐ACTCACCTCTTCAGAACGAATTG‐3’ and 5’‐CCATCTTTGGAAGGTTCAGGTTG‐3’; Human TNF: 5’‐CCTCTCTCTAATCAGCCCTCTG‐3’ and 5’‐GAGGACCTGGGAGTAGATGAG‐3’; Human NOS2: 5’‐TTCAGTATCACAACCTCAGCAAG‐3’ and 5’‐TGGACCTGCAAGTTAAAATCCC‐3’; Human CCL2: 5’‐CAGCCAGATGCAATCAATGCC‐3’ and 5’‐TGGAATCCTGAACCCACTTCT‐3’; Human BAX: 5’‐CCCGAGAGGTCTTTTTCCGAG‐3’ and 5’‐CCAGCCCATGATGGTTCTGAT‐3’; Mouse IL‐1β: 5’‐TCGCAGCAGCACATCAACAAGAG‐3’ and 5’‐AGGTCCACGGGAAAGACACAGG‐3’; Mouse IL‐6: 5’‐CTTCTTGGGACTGATGCTGGTGAC‐3’ and 5’‐AGGTCTGTTGGGAGTGGTATCCTC‐3’; Mouse TNF: 5’‐GCCTCTTCTCATTCCTGCTTGTGG‐3’ and 5’‐GTGGTTTGTGAGTGTGAGGGTCTG‐3’; Mouse NOS2: 5’‐CCAGCGGAGTGACGGCAAAC‐3’ and 5’‐GCAAGACCAGAGGCAGCACATC‐3’; Mouse CCL2: 5’‐CCAGCGGAGTGACGGCAAAC‐3’ and 5’‐GCAAGACCAGAGGCAGCACATC‐3’.

### Immunohistochemistry/Immunofluorescence

This method was modified from a reported protocol.^[^ ^76^ ^]^ Cells were inoculated at a concentration of 1 × 10^4^ per well into 24‐well plates containing cell slides, fixed with paraformaldehyde for 15 min, sealed at room temperature for 30 min, incubated overnight at 4 °C with the appropriate primary antibody, then incubated with secondary antibody and DAPI, and photographed under an upright fluorescence microscope (Leica). Antibodies used in this section as follows: anti‐GFAP (sc‐33673, santa cruz), anti‐Iba1(ab283319, Abcam), anti‐Neun(ab177487, Abcam), anti‐PSD95(ab13552, Abcam), anti‐TMEM119(#90840, Cell Signaling Technology), anti‐MAP2(ab183830, Abcam). The remaining antibodies are described in the western blot section. Embedded mouse brain slices were cut into 6um thick slices using a cryosectioner (Leica) and incubated with the appropriate primary antibody at 4 °C overnight. The slices were then incubated with a fluorescence‐conjugated secondary antibody. The next day, brain slices were incubated with fluorescence‐labelled secondary antibodies and nuclei were stained with DAPI. Images were captured using an upright fluorescence microscope (Leica).

### Bulk Cells RNA‐Seq for Mice and Analysis

First, RNA from the mouse striatum (n = 3) was extracted and the sample was analyzed for integrity and the presence of DNA contamination. Construct RNA libraries using Illumina's NEBNext UltraTM RNA Library Prep Kit. After passing the library check, Illumina sequencing was performed, producing a 150 bp paired‐end reading. Obtain clean data through strict quality control. Hisat2 was selected as the mapping tool, and the splice‐linked database was constructed according to the gene model annotation file. After gaining the expression matrix, we processed the data for characterization. The DESeq2R software package (1.16.1) was used for differential expression analysis. The ggplot2 R package was applied to plot the differential genes into volcano plots based on their p‐value and fold change, and both Wald test and Benjamini‐Hochberg method were adopted for these analysis. The Mfuzz R package was used to fuzzy cluster the trend changes of the genes across treatment groups to seek for gene clusters that were reversed by NMN. Advanced analyses were carried out after finding the significant clusters of genes. GO enrichment analysis of differentially expressed genes was performed using the ClusterProfiler R software package. GSEA was implemented by the ClusterProfiler R package and analyzed via permutation tests and the Benjamini‐Hochberg method. PPI analysis based on the differentially expressed genes was performed according to the STRING database.

### Sample Preparation and Metabolomics Analysis

After being obtained, the striatum was snap‐frozen for subsequent metabolomic analysis. Then, a UHPLC (1290 Infinity LC, Agilent Technologies) coupled to a quadrupole time‐of‐flight instrument (AB Sciex TripleTOF 6600) was applied to analyze these samples for further untargeted metabolomics. In the positive and negative ionization mode, both mobile phase A contained an aqueous solution of 25 mM ammonium acetate and 25 mM ammonium hydroxide, while both mobile phase B included acetonitrile. The gradient elution was manipulated and followed by first 85% B for 1 min, then dropping to 65% in 11 min, reaching 40% in 0.1 min, maintaining it for 4 min, and finally raising it to 85% B in 0.1 min. During the MS acquisition, the m/z range of the instrument was selected as 60 ‐ 1000 Da, while the accumulation time of the TOF MS scan was set at 0.20 s/spectrum. Ion peaks were compared to standards in the reference library for identifying the structure and quantifying the relative levels. Following this, the data was filtered for empty values, populated by KNN, and filtered for features with an RSD > 50%, and subsequent QC was then performed, with the results being utilized for further analyses. R packages including DESeq2R, ggplot2, and mfuzz were used for metabolite characterization and both Wald test and Benjamini‐Hochberg method were adopted for these analysis. The R package was employed for Orthogonal Partial least squares Discriminant Analysis (OPLS‐DA), and Permutation Test was applied for OPLS‐DA. Moreover, for advanced analysis, MSEA was analyzed based on the R package provided by MetaboAnalyst, and the Hypergeometric Test was applied. Also, weighted gene co‐expression network analysis (WCGNA) based on the WCGNA R package was also further carried out to identify modules for co‐expression of metabolites associated with NMN, and analyzed using Pearson correlation coefficients, permutation tests, as well as t‐tests to assess the correlation coefficients, module‐phenotype associations, and their significance.

### Participants and Neuropsychological Measurements

The cohort consisted of clinically diagnosed PD participants and age‐matched healthy controls (HC) from the Wenzhou Medical University of First Affiliated Hospital, between March 2018 and May 2024. This study was approved by the Institutional Ethics Committee of the First Affiliated Hospital of Wenzhou Medical University (KY2021‐153). All participants provided informed consent before enrolment. Only plasma data were collected from Cohort A (HC = 76, PD = 78) and Cohort B (HC = 46, PD = 50) participants, while both plasma and ^18^F‐FP‐CIT‐PET data were collected from Cohort C (PD = 40). Motor symptoms and progression stage of PD were assessed in participants with recruited PD who met the MDS clinical diagnostic criteria,^[^ ^77^ ^]^ Unified Parkinson's Disease Rating Scale (UPDRS), and Hoehn‐Yahr staging. Additional baseline data were used to assess cognitive and emotional aspects, including the Chinese version of the Mini‐Mental State Examination (MMSE), the Hamilton Depression Rating Scale‐17 items (HAMD), and the Hamilton Anxiety Rating Scale (HAMA).

### Measurement of Plasma Biomarkers

Blood samples were collected using EDTA anticoagulant tubes and centrifuged at 3000 rpm for 10 min within 1 h of collection to obtain plasma. Plasma samples were stored at −80 °C until further analysis. The levels of ATF4, ATF5, CHOP, PINK1, and Parkin in the plasma were measured using enzyme‐linked immunosorbent assays (ELISA). Continuous variables were assessed for normality using the Kolmogorov‐Smirnov test, histograms, and Q‐Q plots. Data were expressed as mean (standard deviation, SD). Group comparisons were conducted using analysis of covariance (ANCOVA) or Kruskal‐Wallis tests, followed by Bonferroni correction for multiple comparisons. Statistical analyses were performed using R software (v 4.3.1) and SPSS (v29.0.1.0). Statistical significance was set at a two‐tailed *p*‐value of less than 0.05.

### PET‐Imaging Procedures Analysis

DAT scans were obtained using an ^18^F‐FP‐CIT PET‐CT scanner (PHILIPS). DAT images were acquired 90 min after injection during a 20‐min session in 3D mode.^[^ ^78^ ^]^ 2.3 mm full‐width half‐maximum 3D Gaussian smoothing was performed post hoc. Six regions of interest (ROIs) were defined: left caudate (L‐caudate), right caudate (R‐caudate), left anterior putamen (L‐AP), right anterior putamen (R‐AP), left posterior putamen (L‐PP) and right posterior putamen (R‐PP). To obtain the standardized uptake value ratio (SUVR), the standardized uptake value (SUV) for each ROI was normalized to the mean SUV of the cerebellar region. The correlation between plasma UPR^mt^ biomarkers and SUV mean values was assessed. Pearson correlation analysis was used for normally distributed variables. For non‐normally distributed variables, Spearman correlation analysis was used.

### Statistical Analysis

This work used a two‐tailed unpaired *t*‐test for comparison between two groups, or One‐Way ANOVA for comparison among multiple groups. All data were presented as mean ± S.E.M. as indicated with *p value < 0.05 considered statistically significant. The Pearson correlation coefficient was applied to assess the linear relationship between two variables, and subsequently, simple linear regression was employed to determine the parameters of the linear equation. Data were plotted and analyzed with GraphPad Prism 8.0 software (GraphPad Software) and IBM SPSS Statistics 26.

### Ethics Approval and Consent to Participate

The study was approved by the institutional Ethics Board Committee of the Wenzhou Medical University First Affiliated Hospital (KY2021‐153). All participants provided written informed consent before participating in this study.

## Conflict of Interest

The authors declare no conflict of interest.

## Author Contributions

S.Z., X.X., and J.H. contributed equally to this work. X.S.W., W.H.S., and C.L.X. designed and coordinated the project. X.X., S.T.Z., J.L.H., and J.N.H. did the cell experiments. Q.D., H,J,C., H.J.H., J.L.C., K.K.C. and Y.Z. helped with data analysis on neurodegeneration. J.X.X and S.T.Z. did the mouse experiments. X.S.W., C.L.X., and S.T.Z. wrote the manuscript, while W.W.W., J.J.Q. and K.P. edited the manuscript.

## Supporting information



Supporting Information

## Data Availability

The data that support the findings of this study are available on request from the corresponding author. The data are not publicly available due to privacy or ethical restrictions.

## References

[1] B. R. Bloem , M. S. Okun , C. Klein , Lancet 2021, 397, 2284. 33848468 10.1016/S0140-6736(21)00218-X

[2] G. Lawrence , C. L. Holley , K. Schroder , Trends Immunol. 2022, 43, 877. 36229358 10.1016/j.it.2022.09.010

[3] A. H. Schapira , J. M. Cooper , D. Dexter , P. Jenner , J. B. Clark , C. D. Marsden , Lancet 1989, 1, 1269. 2566813 10.1016/s0140-6736(89)92366-0

[4] A. T. Moehlman , R. J. Youle , Annu. Rev. Cell Dev. Biol. 2020, 36, 265. 33021820 10.1146/annurev-cellbio-021820-101354

[5] A. H. Schapira , Lancet Neurol. 2008, 7, 97. 18093566 10.1016/S1474-4422(07)70327-7

[6] M. A. Eldeeb , R. A. Thomas , M. A. Ragheb , A. Fallahi , E. A. Fon , Physiol. Rev. 2022, 102, 1721. 35466694 10.1152/physrev.00041.2021

[7] S. Lautrup , D. A. Sinclair , M. P. Mattson , E. F. Fang , Cell Metab. 2019, 30, 630. 31577933 10.1016/j.cmet.2019.09.001 PMC6787556

[8] T. E. S. Kauppila , J. H. K. Kauppila , N. G. Larsson , Cell Metab. 2017, 25, 57. 28094012 10.1016/j.cmet.2016.09.017

[9] C. Cantó , K. J. Menzies , J. Auwerx , Cell Metab. 2015, 22, 31. 26118927 10.1016/j.cmet.2015.05.023 PMC4487780

[10] E. F. Fang , M. Scheibye‐Knudsen , L. E. Brace , H. Kassahun , T. SenGupta , H. Nilsen , J. R. Mitchell , D. L. Croteau , V. A. Bohr , Cell 2014, 157, 882. 24813611 10.1016/j.cell.2014.03.026 PMC4625837

[11] K. Kaarniranta , H. Uusitalo , J. Blasiak , S. Felszeghy , R. Kannan , A. Kauppinen , A. Salminen , D. Sinha , D. Ferrington , Prog. Retinal Eye Res. 2020, 79, 100858. 10.1016/j.preteyeres.2020.100858 PMC7650008 32298788

[12] C. Münch , J. W. Harper , Nature 2016, 534, 710. 27350246 10.1038/nature18302 PMC4939261

[13] F. X. R. Sutandy , I. Gößner , G. Tascher , C. Münch , Nature 2023, 618, 849. 37286597 10.1038/s41586-023-06142-0 PMC10284689

[14] A. Melber , C. M. Haynes , Cell Res. 2018, 28, 281. 29424373 10.1038/cr.2018.16 PMC5835775

[15] B. Xiao , J. Kuruvilla , E. K. Tan , NPJ Parkinsons Dis 2022, 8, 135. 36257956 10.1038/s41531-022-00402-y PMC9579202

[16] R. J. Youle , D. P. Narendra , Nat. Rev. Mol. Cell Biol. 2011, 12, 9. 21179058 10.1038/nrm3028 PMC4780047

[17] S. L. Sison , A. D. Ebert , Aging (Albany NY) 2018, 10, 526. 29706611 10.18632/aging.101433 PMC5940114

[18] D. C. Schöndorf , D. Ivanyuk , P. Baden , A. Sanchez‐Martinez , S. De Cicco , C. Yu , I. Giunta , L. K. Schwarz , G. Di Napoli , V. Panagiotakopoulou , S. Nestel , M. Keatinge , J. Pruszak , O. Bandmann , B. Heimrich , T. Gasser , A. J. Whitworth , M. Deleidi , Cell Rep. 2018, 23, 2976. 29874584 10.1016/j.celrep.2018.05.009

[19] K. Essuman , D. W. Summers , Y. Sasaki , X. Mao , A. DiAntonio , J. Milbrandt , Neuron 2017, 93, 1334. 28334607 10.1016/j.neuron.2017.02.022 PMC6284238

[20] T. I. Kam , X. Mao , H. Park , S. C. Chou , S. S. Karuppagounder , G. E. Umanah , S. P. Yun , S. Brahmachari , N. Panicker , R. Chen , S. A. Andrabi , C. Qi , G. G. Poirier , O. Pletnikova , J. C. Troncoso , L. M. Bekris , J. B. Leverenz , A. Pantelyat , H. S. Ko , L. S. Rosenthal , T. M. Dawson , V. L. Dawson , Science 2018, 362, 6414. 10.1126/science.aat8407 PMC6431793 30385548

[21] M. J. Pérez , P. Baden , M. Deleidi , Mech. Ageing Dev. 2021, 197, 111499. 33989633 10.1016/j.mad.2021.111499

[22] E. F. Fang , S. Lautrup , Y. Hou , T. G. Demarest , D. L. Croteau , M. P. Mattson , V. A. Bohr , Trends Mol. Med. 2017, 23, 899 28899755 10.1016/j.molmed.2017.08.001 PMC7494058

[23] Q. Zheng , H. Liu , H. Zhang , Y. Han , J. Yuan , T. Wang , Y. Gao , Z. Li , Adv. Sci. (Weinh) 2023, 10, 2300758. 37202595 10.1002/advs.202300758 PMC10401119

[24] Z. D. Zhou , E. K. Tan , Ageing Res. Rev. 2020, 62, 101107. 32535274 10.1016/j.arr.2020.101107

[25] J. Roboon , T. Hattori , H. Ishii , M. Takarada‐Iemata , D. T. Nguyen , C. D. Heer , D. O'Meally , C. Brenner , Y. Yamamoto , H. Okamoto , H. Higashida , O. Hori , J. Neurochem. 2021, 158, 311. 33871064 10.1111/jnc.15367 PMC8282715

[26] Y. Zhu , C. Li , X. Tao , J. M. Brazill , J. Park , Z. Diaz‐Perez , R. G. Zhai , *Proc. Natl. Acad. Sci*. USA 2019, 116, 19165. 10.1073/pnas.1904563116 PMC6754563 31484760

[27] A. Perelman , C. Wachtel , M. Cohen , S. Haupt , H. Shapiro , A. Tzur , Cell Death Dis. 2012, 3, 430. 10.1038/cddis.2012.171 PMC3542606 23171850

[28] G. M. Fogo , S. Raghunayakula , K. J. Emaus , F. J. Torres Torres , J. M. Wider , T. H. Sanderson , FASEB J. 2024, 38, 70066. 10.1096/fj.202400313R PMC11542587 39312414

[29] A. C. Hijam , Y. C. Tongbram , P. D. Nongthombam , H. N. Meitei , A. S. Koijam , Y. Rajashekar , R. Haobam , J. Ethnopharmacol. 2024, 330, 118197. 38636579 10.1016/j.jep.2024.118197

[30] A. Lin , C. J. Giuliano , A. Palladino , K. M. John , C. Abramowicz , M. L. Yuan , E. L. Sausville , D. A. Lukow , L. Liu , A. R. Chait , Z. C. Galluzzo , C. Tucker , J. M. Sheltzer , Sci. Transl. Med. 2019, 11, 509. 10.1126/scitranslmed.aaw8412 PMC7717492 31511426

[31] S. Qian , H. He , X. Xiong , R. Ai , W. Wang , H. Zhu , Q. Ye , S. Zhou , H. Nilsen , C. Xie , CNS Neurosci. Ther. 2024, 30, 14532. 10.1111/cns.14532 PMC11056850 37990436

[32] D. Cossu , K. Yokoyama , L. A. Sechi , N. Hattori , Front. Immunol. 2021, 12, 681386. 34421896 10.3389/fimmu.2021.681386 PMC8371632

[33] Y. F. Lin , A. M. Schulz , M. W. Pellegrino , Y. Lu , S. Shaham , C. M. Haynes , Nature 2016, 533, 7603. 10.1038/nature17989 PMC4873342 27135930

[34] J. Liu , X. He , S. Zheng , A. Zhu , J. Wang , Oxid. Med. Cell. Longevity 2022, 2022, 6430342. 10.1155/2022/6430342 PMC9519344 36187338

[35] I. Lee , J. S. Kim , J. Y. Park , B. H. Byun , S. Y. Park , J. H. Choi , H. Moon , J. Y. Kim , K. C. Lee , D. Y. Chi , K. M. Kim , I. Lim , J. H. Kang , S. H. Ahn , B. I. Kim , J. H. Ha , S. M. Lim , Synapse 2018, 72, 22032. 10.1002/syn.22032 29486515

[36] P. Gowda , P. H. Reddy , S. Kumar , Ageing Res. Rev. 2022, 73, 101529. 34813976 10.1016/j.arr.2021.101529 PMC8692431

[37] H. Zhang , D. Ryu , Y. Wu , K. Gariani , X. Wang , P. Luan , D. D'Amico , E. R. Ropelle , M. P. Lutolf , R. Aebersold , K. Schoonjans , K. J. Menzies , J. Auwerx , Science 2016, 352, 1436. 27127236 10.1126/science.aaf2693

[38] F. J. Bock , S. W. G. Tait , Nat. Rev. Mol. Cell Biol. 2020, 21, 85. 31636403 10.1038/s41580-019-0173-8

[39] N. W. Dunham , T. S. Miya , J. Am. Pharm. Assoc. 1957, 46, 208. 10.1002/jps.3030460322 13502156

[40] Q. Wang , Y. Liu , J. Zhou , Transl. Neurodegener. 2015, 4, 19. 26464797 10.1186/s40035-015-0042-0 PMC4603346

[41] S. F. Soukup , R. Vanhauwaert , P. Verstreken , EMBO J. 2018, 37, 18. 10.15252/embj.201898960 PMC6138432 30065071

[42] M. Nguyen , Y. C. Wong , D. Ysselstein , A. Severino , D. Krainc , Trends Neurosci. 2019, 42, 140. 30509690 10.1016/j.tins.2018.11.001 PMC6452863

[43] A. M. Pickrell , R. J. Youle , Neuron 2015, 85, 257. 25611507 10.1016/j.neuron.2014.12.007 PMC4764997

[44] S. G. Dastidar , M. T. Pham , M. B. Mitchell , S. G. Yeom , S. Jordan , A. Chang , B. L. Sopher , A. R. La Spada , J. Neurosci. 2020, 40, 8734. 33046555 10.1523/JNEUROSCI.0940-20.2020 PMC7643294

[45] L. Mouchiroud , R. H. Houtkooper , N. Moullan , E. Katsyuba , D. Ryu , C. Cantó , A. Mottis , Y. S. Jo , M. Viswanathan , K. Schoonjans , L. Guarente , J. Auwerx , Cell 2013, 154, 430. 23870130 10.1016/j.cell.2013.06.016 PMC3753670

[46] M. S. Bonkowski , D. A. Sinclair , Nat. Rev. Mol. Cell Biol. 2016, 17, 679. 27552971 10.1038/nrm.2016.93 PMC5107309

[47] K. Veverová , J. Laczó , A. Katonová , H. Horáková , V. Matušková , F. Angelucci , M. Laczó , Z. Nedelská , J. Hort , H. L. Wang , J. Zhang , L. Shi , E. Fei Fang , M. Vyhnálek , Autophagy 2024, 20, 1868. 38695174 10.1080/15548627.2024.2340408 PMC11262225

[48] D. Hu , Z. Liu , X. Qi , Biochem. Biophys. Res. Commun. 2021, 569, 17. 34216993 10.1016/j.bbrc.2021.06.079 PMC8364872

[49] J. F. Cooper , E. Machiela , D. J. Dues , K. K. Spielbauer , M. M. Senchuk , J. M. Van Raamsdonk , Sci. Rep. 2017, 7, 16441. 29180793 10.1038/s41598-017-16637-2 PMC5703891

[50] D. Hu , X. Sun , X. Liao , X. Zhang , S. Zarabi , A. Schimmer , Y. Hong , C. Ford , Y. Luo , X. Qi , Acta Neuropathol. 2019, 137, 939. 30877431 10.1007/s00401-019-01993-2 PMC6531426

[51] E. H. Clark , A. Vázquez de la Torre , T. Hoshikawa , T. Briston , J. Biol. Chem. 2021, 296, 100209. 33372898 10.1074/jbc.REV120.014294 PMC7948953

[52] A. B. Malpartida , M. Williamson , D. P. Narendra , R. Wade‐Martins , B. J. Ryan , Trends Biochem. Sci. 2021, 46, 329. 33323315 10.1016/j.tibs.2020.11.007

[53] E. M. Valente , P. M. Abou‐Sleiman , V. Caputo , M. M. Muqit , K. Harvey , S. Gispert , Z. Ali , D. Del Turco , A. R. Bentivoglio , D. G. Healy , A. Albanese , R. Nussbaum , R. González‐Maldonado , T. Deller , S. Salvi , P. Cortelli , W. P. Gilks , D. S. Latchman , R. J. Harvey , B. Dallapiccola , G. Auburger , N. W. Wood , Science 2004, 304, 1158. 15087508 10.1126/science.1096284

[54] T. I. Kam , J. T. Hinkle , T. M. Dawson , V. L. Dawson , Neurobiol. Dis. 2020, 144, 105028. 32736085 10.1016/j.nbd.2020.105028 PMC7484088

[55] Y. Hou , Y. Wei , S. Lautrup , B. Yang , Y. Wang , S. Cordonnier , M. P. Mattson , D. L. Croteau , V. A. Bohr , *Proc. Natl. Acad. Sci*. USA 2021, 118, 37. 10.1073/pnas.2011226118 PMC8449423 34497121

[56] H. R. Li , Q. Liu , C. L. Zhu , X. Y. Sun , C. Y. Sun , C. M. Yu , P. Li , X. M. Deng , J. F. Wang , Redox Biol. 2023, 63, 102745. 37201414 10.1016/j.redox.2023.102745 PMC10206198

[57] Y. Hou , S. Lautrup , S. Cordonnier , Y. Wang , D. L. Croteau , E. Zavala , Y. Zhang , K. Moritoh , J. F. O'Connell , B. A. Baptiste , T. V. Stevnsner , M. P. Mattson , V. A. Bohr , *Proc. Natl. Acad. Sci*. USA 2018, 115, E1876. 10.1073/pnas.1718819115 PMC5828618 29432159

[58] J. Yoshino , J. A. Baur , S. I. Imai , Cell Metab. 2018, 27, 513. 29249689 10.1016/j.cmet.2017.11.002 PMC5842119

[59] X. Wang , H. J. He , X. Xiong , S. Zhou , W. W. Wang , L. Feng , R. Han , C. L. Xie , Front. Cell Dev. Biol. 2021, 9, 668491. 34414179 10.3389/fcell.2021.668491 PMC8369418

[60] K. F. Mills , S. Yoshida , L. R. Stein , A. Grozio , S. Kubota , Y. Sasaki , P. Redpath , M. E. Migaud , R. S. Apte , K. Uchida , J. Yoshino , S. I. Imai , Cell Metab. 2016, 24, 795. 28068222 10.1016/j.cmet.2016.09.013 PMC5668137

[61] J. Irie , E. Inagaki , M. Fujita , H. Nakaya , M. Mitsuishi , S. Yamaguchi , K. Yamashita , S. Shigaki , T. Ono , H. Yukioka , H. Okano , Y. I. Nabeshima , S. I. Imai , M. Yasui , K. Tsubota , H. Itoh , Endocr. J. 2020, 67, 153. 31685720 10.1507/endocrj.EJ19-0313

[62] L. E. I. Lu , L. E. Tang , W. Wei , Y. Hong , H. Chen , W. Ying , S. Chen , Exp. Ther. Med. 2014, 8, 943. 25120628 10.3892/etm.2014.1842 PMC4113526

[63] L. Mouchiroud , R. H. Houtkooper , N. Moullan , E. Katsyuba , D. Ryu , C. Cantó , A. Mottis , Y.‐S. Jo , M. Viswanathan , K. Schoonjans , L. Guarente , J. Auwerx , Cell 2013, 154, 430. 23870130 10.1016/j.cell.2013.06.016 PMC3753670

[64] L. Yang , Z. Ruan , X. Lin , H. Wang , Y. Xin , H. Tang , Z. Hu , Y. Zhou , Y. Wu , J. Wang , D. Qin , G. Lu , K. M. Loomes , W.‐Y. Chan , X. Liu , Nat. Commun. 2024, 15, 1. 38228611 10.1038/s41467-024-44808-z PMC10791663

[65] X. Han , B. Han , Y. Zhao , G. Li , T. Wang , J. He , W. Du , X. Cao , J. Gan , Z. Wang , W. Zheng , Nutrients 2022, 14, 17. 10.3390/nu14173508 PMC9460683 36079767

[66] H. Su , F. Yang , R. Fu , X. Li , R. French , E. Mose , X. Pu , B. Trinh , A. Kumar , J. Liu , L. Antonucci , J. Todoric , Y. Liu , Y. Hu , M. T. Diaz‐Meco , J. Moscat , C. M. Metallo , A. M. Lowy , B. Sun , M. Karin , Cancer Cell 2021, 39, 678. 33740421 10.1016/j.ccell.2021.02.016 PMC8119368

[67] C. Yu , D. Luo , J. Yu , M. Zhang , X. Zheng , G. Xu , J. Wang , H. Wang , Y. Xu , K. Jiang , J. Xu , X. Ma , J. Jing , H. Shi , Oncogene 2022, 41, 191. 34718347 10.1038/s41388-021-02077-w PMC8732282

[68] D. W. Luchtman , D. Shao , C. Song , Physiol. Behav. 2009, 98, 130. 19410592 10.1016/j.physbeh.2009.04.021

[69] W. Zhang , S. Chen , X. Huang , H. Tong , H. Niu , L. Lu , Cell Death Discovery 2023, 9, 251. 37460539 10.1038/s41420-023-01549-0 PMC10352270

[70] H. Kuribara , Y. Higuchi , S. Tadokoro , Jpn J. Pharmacol. 1977, 27, 117. 864872 10.1254/jjp.27.117

[71] Y. Liu , H. Liu , Y. Lu , X. Yin , W. Lu , X. Lian , K. Wang , C. Shi , Z. Yao , J. F. Chen , Z. Li , Exp. Neurol. 2023, 364, 114396. 37003486 10.1016/j.expneurol.2023.114396

[72] Y. Liu , H. Liu , Y. Lu , X. Yin , W. Lu , X. Lian , K. Wang , C. Shi , Z. Yao , J. F. Chen , Z. Li , Exp. Neurol. 2023, 364, 114396. 37003486 10.1016/j.expneurol.2023.114396

[73] C. Li , B. Wu , Y. Li , J. Chen , Z. Ye , X. Tian , J. Wang , X. Xu , S. Pan , Y. Zheng , X. Cai , L. Jiang , M. Zhao , Cell Stem Cell 2022, 29, 1119. 35803229 10.1016/j.stem.2022.06.004

[74] X. Zhang , X. Zuo , B. Yang , Z. Li , Y. Xue , Y. Zhou , J. Huang , X. Zhao , J. Zhou , Y. Yan , H. Zhang , P. Guo , H. Sun , L. Guo , Y. Zhang , X. D. Fu , Cell 2014, 158, 607. 25083871 10.1016/j.cell.2014.05.047 PMC4119298

[75] H. Y. Jiang , Y. Yang , Y. Y. Zhang , Z. Xie , X. Y. Zhao , Y. Sun , W. J. Kong , Redox Biol. 2018, 14, 361. 29049980 10.1016/j.redox.2017.10.002 PMC5641921

[76] X. Liu , L. Qu , N. Zhang , X. Yu , Z. Xiao , L. Song , J. Xie , H. Xu , Front. Mol. Neurosci. 2020, 13, 613404. 33469419 10.3389/fnmol.2020.613404 PMC7813998

[77] D. Berg , R. B. Postuma , C. H. Adler , B. R. Bloem , P. Chan , B. Dubois , T. Gasser , C. G. Goetz , G. Halliday , L. Joseph , A. E. Lang , I. Liepelt‐Scarfone , I. Litvan , K. Marek , J. Obeso , W. Oertel , C. W. Olanow , W. Poewe , M. Stern , G. Deuschl , Mov. Disord. 2015, 30, 1600. 26474317 10.1002/mds.26431

[78] H. S. Yoo , S. J. Chung , S. J. Kim , J. S. Oh , J. S. Kim , B. S. Ye , Y. H. Sohn , P. H. Lee , Eur. J. Nucl. Med. Mol. Imaging 2018, 45, 1585. 29728749 10.1007/s00259-018-4019-y

