# Supplementary material for: NAD+‐Boosters Improve Mitochondria Quality Control In Parkinson's Disease Models Via Mitochondrial UPR
Source: Adv Sci (Weinh). 2025 Jul 20;12(38):e08503. doi: 10.1002/advs.202408503 (PMC12520555; doi:10.1002/advs.202408503)

ADVANCED SCIENCE
Open Access

Supporting Information

for *Adv. Sci.*, DOI 10.1002/advs.202408503

NAD+-Boosters Improve Mitochondria Quality Control In Parkinson's Disease Models Via Mitochondrial UPR

Shuoting Zhou, Xi Xiong, Jialong Hou, Qi Duan, Yi Zheng, Tao Jiang, Jiani Huang, Haijun He, Jiaxue Xu, Keke Chen, Wenwen Wang, Jinlai Cai, Jingjing Qian, Huijun Chen, Weihong Song, XinShi Wang* and Chenglong Xie**

Supplementary documents

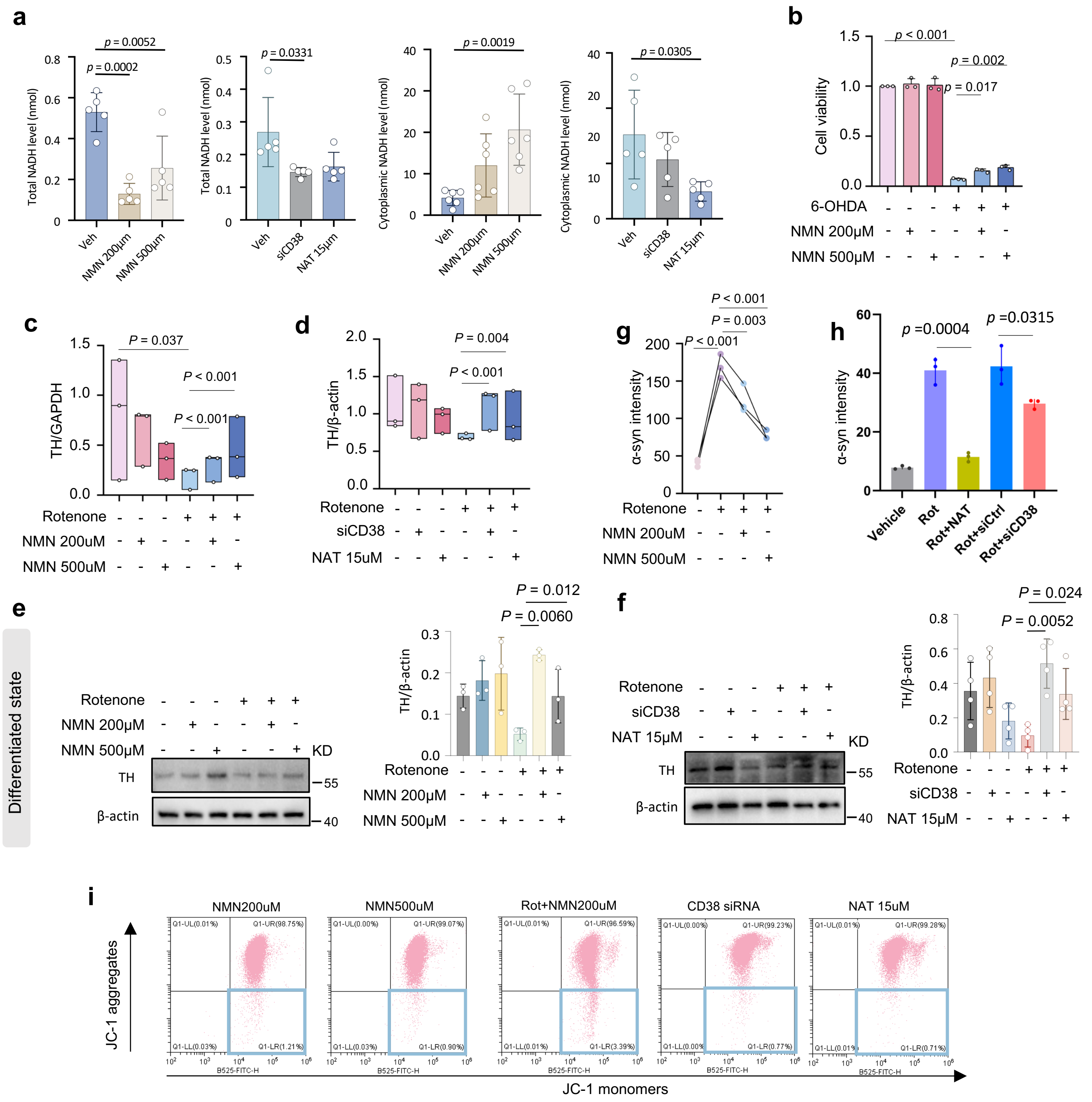

Supple Figure 1

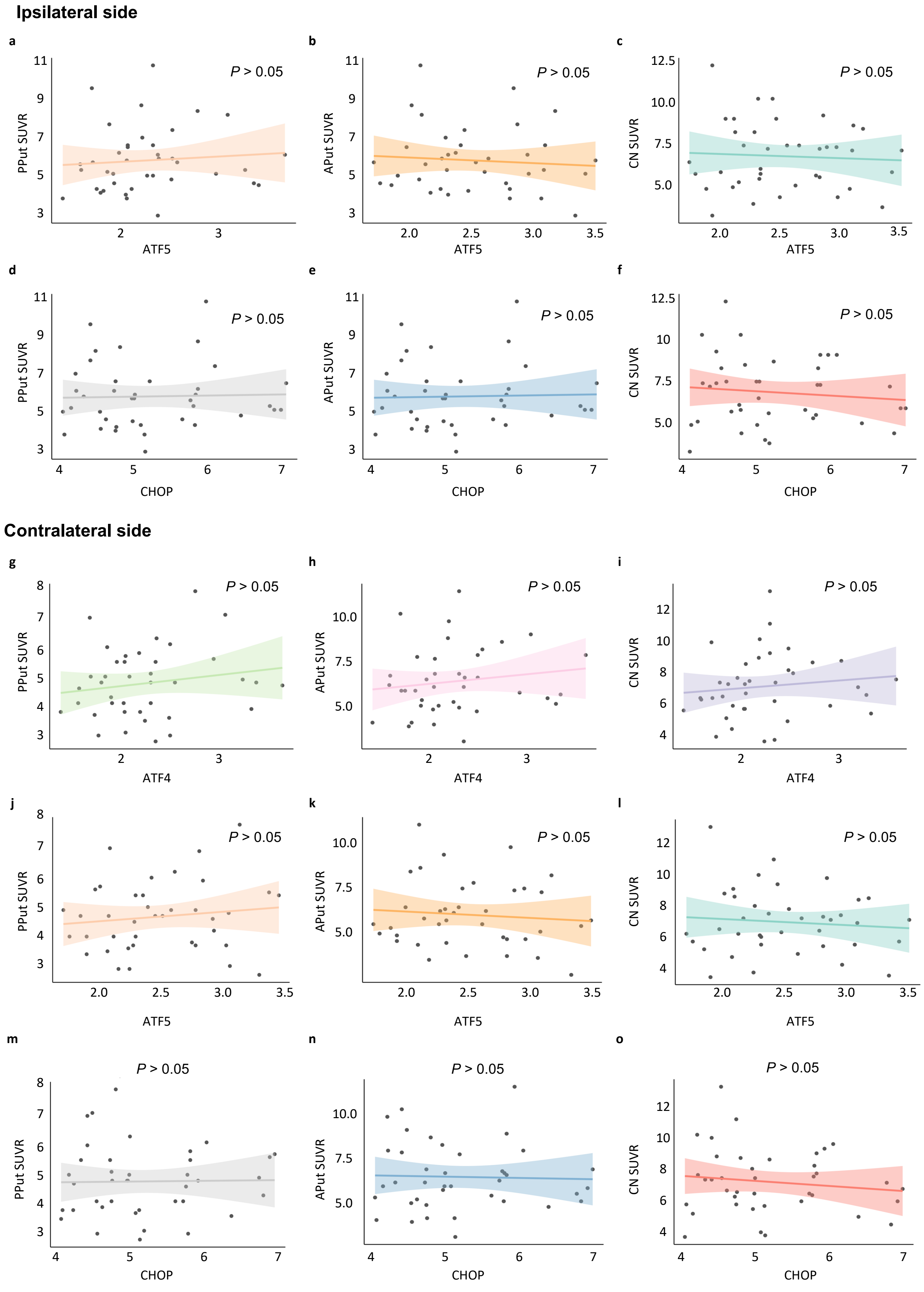

Supple Figure 2

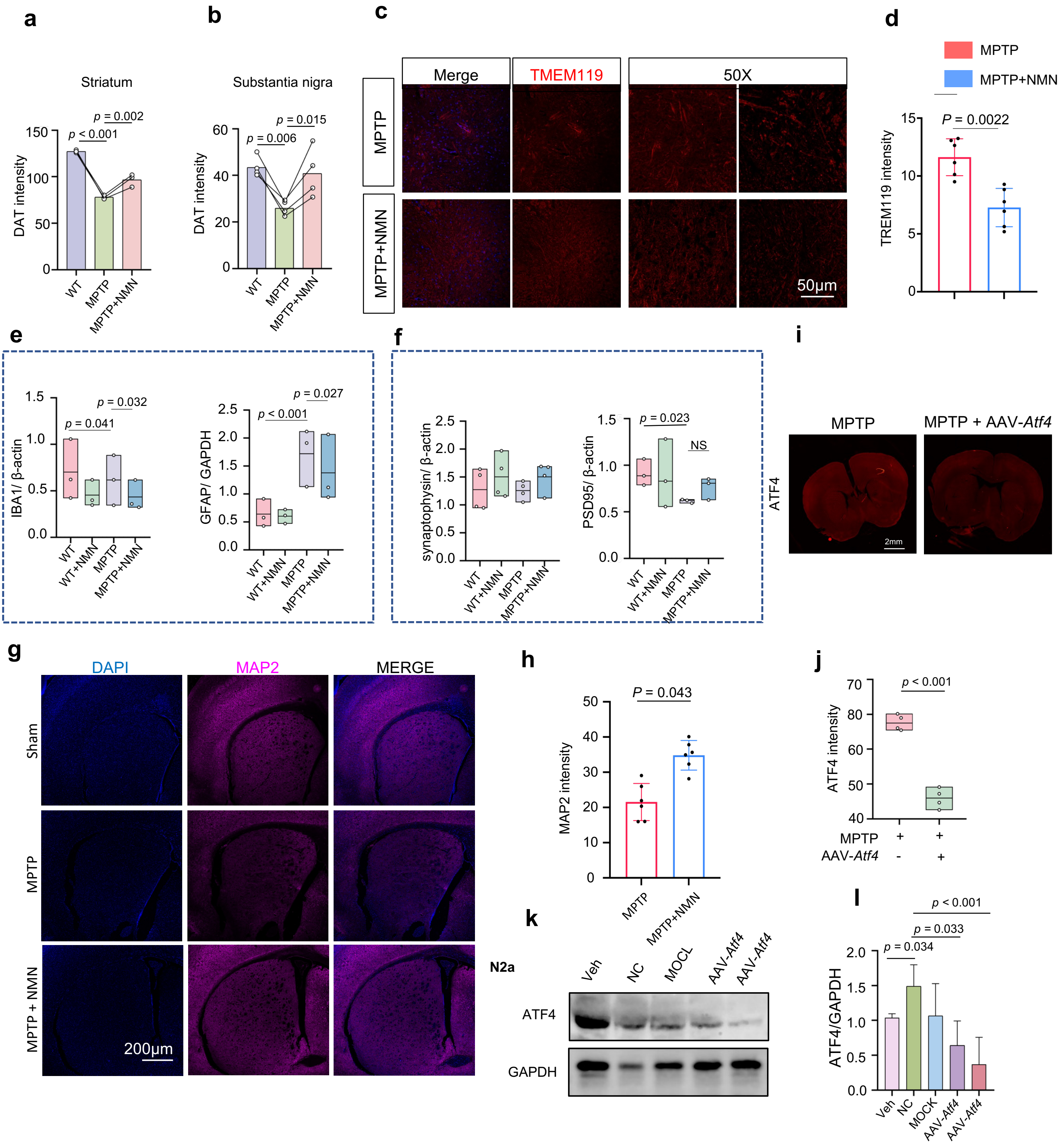

Supple Figure 3

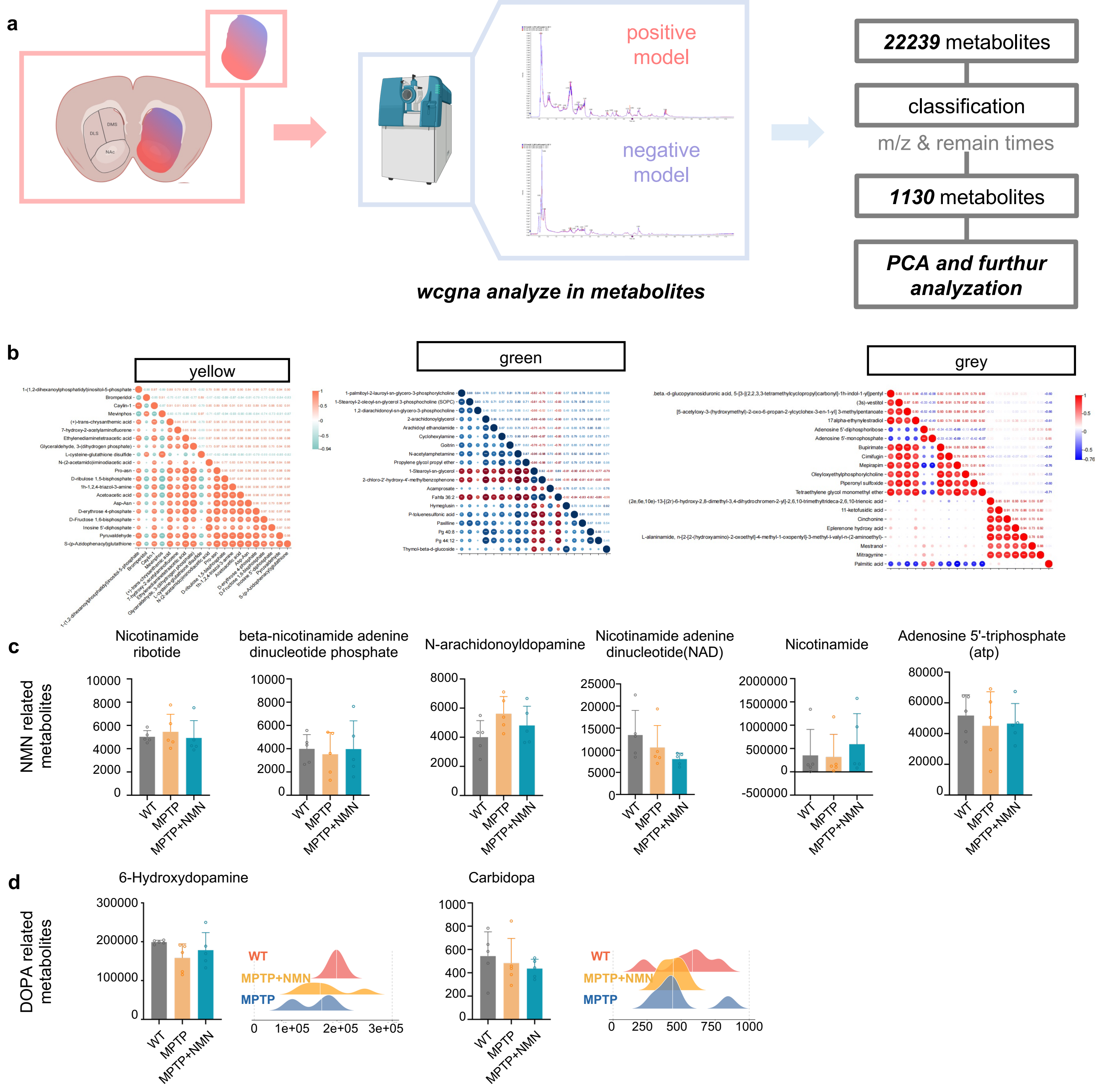

Supple Figure 4

Supple Table 1: Basic demographic characteristics of the cohort

	Cohort a			Cohort b			Cohort c
	HC	PD		HC	PD		PD
	N=75	N=78	P value	N=46	N=49	P value	N=40
Age	63.9 (8.42)	66.5 (10.5)	0.084	63.8 (9.19)	70.8 (7.64)	<0.001	1320 (83.0)
Gender			0.219			0.338	
Female	44 (58.7%)	37 (47.4%)		28 (60.9%)	24 (49.0%)		52 (54.7%)
Male	31 (41.3%)	41 (52.6%)		18 (39.1%)	25 (51.0%)		43 (45.2%)
Height	162 (7.23)	162 (7.91)	0.776	162 (8.06)	161 (7.58)	0.513	0.600 (0.496)
Weight	63.5 (9.36)	61.7 (9.65)	0.27	63.9 (9.46)	58.2 (13.1)	0.018	163 (6.56)
BMI	24.2 (2.98)	23.3 (4.13)	0.132	24.3 (3.01)	22.5 (4.52)	0.02	64.9 (10.0)
Education	4.53 (4.68)	4.43 (3.87)	0.887	4.63 (5.12)	4.00 (3.32)	0.482	24.2 (2.61)
Disease history	0.78 (2.81)	3.19 (2.86)	<0.001	1.29 (3.75)	3.44 (3.00)	0.003	5.30 (4.42)
UPDRS		39.0 (20.2)			44.1 (20.5)		2.36 (1.90)
UPDRS I		1.77 (1.83)			2.16 (1.94)		28.2 (13.2)
UPDRS II		11.8 (13.7)			13.7 (16.5)		1.23 (1.83)
UPDRS III		25.7 (13.8)			28.8 (14.1)		7.53 (4.44)
UPDRS IV		1.15 (1.77)			1.08 (1.63)		18.5 (9.22)
MMSE	23.9 (4.21)	20.9 (6.77)	0.002	24.1 (4.45)	19.8 (6.99)	0.001	2.11 (3.07)
HAMD	3.28 (3.49)	5.27 (4.84)	0.004	3.35 (3.91)	5.88 (4.54)	0.004	22.5 (6.50)
HAMA	4.65 (4.30)	7.54 (5.60)	<0.001	4.22 (4.47)	8.73 (5.75)	<0.001	4.25 (5.63)
RBD	7.29 (9.59)	15.8 (18.0)	<0.001	6.48 (8.68)	16.9 (17.6)	<0.001	6.18 (7.09)
ADL	22.6 (13.0)	30.7 (12.0)	<0.001	24.3 (16.5)	33.4 (12.6)	0.003	13.6 (19.1)

PD: Parkinson's disease; HC: Healthy Control; BMI: Body Mass Index; UPDRS: unified Parkinson's disease rating scale; MMSE: Mini-Mental State Examination; HAMD: Hamilton Depression Scale; HAMA: Hamilton Anxiety Scale; RBDQ-HK: REM sleep behavior Disorder questionnaire-Hong Kong; ADL: Activity of Daily Living Scale.

Supple Table 2. Plasma Levels of UPR^{mt} and mitophagy of participants in Cohort a and b

Biomarker (ng/mL)	Minimum		Maximun		Mean		SD		95%CI	P vaule
	HC	PD	HC	PD	HC	PD	HC	PD		
ATF4[a]	0.65	1.38	6.6705	8.787	3.5535	5.0471	2.0058	2.6115	[-2.0984, -0.88006]	P<0.0001
ATF5[a]	0.79	1.7	7.4814	9.7454	4.122	5.5649	2.2233	2.7544	[-2.12, -0.79868]	P<0.0001
CHOP[a]	2.58	3.89	12.479	15.894	7.1195	9.443	2.9473	4.0072	[-3.4053, -1.16]	P<0.0001
PINK1[b]	48.915	60.423	82.183	112.64	66.772	87.016	9.0379	13.988	[15.502, 24.987]	P<0.0001
Parkin[b]	11.637	13.406	19.616	25.544	15.735	20.029	2.2468	3.2941	[3.1578, 5.4301]	P<0.0001

Cohort a: HC=76, PD=78; Cohort b: HC=46, PD=49

Supple Table 3: ROC analysis results of UPR^{mt} and mitophagy biomarkers for PD vs HC

Biomarker (ng/mL)	AUC	95% (CI)	Cut-off value	Sensitivity	Specificity	Accuracy	Youden Index
ATF4[a]	0.717	0.635 - 0.799	5.9353	0.90789	0.51282	0.70779	0.42072
ATF5[a]	0.703	0.620 - 0.786	7.2403	0.98684	0.42308	0.7013	0.40992
CHOP[a]	0.697	0.614 - 0.780	11.789	0.97368	0.37179	0.66883	0.34548
PINK1[b]	0.889	0.825 - 0.953	76.225	0.78	0.86957	0.82292	0.64957
Parkin[b]	0.848	0.773 - 0.923	18.7	0.64	0.91304	0.77083	0.55304

95% CI: 95% Confidence Interval.

Supple Table 4. SUVR values of PET-CT in severe side brain regions (CN, AP, and PP) for Plasma UPR^{mt} Biomarker in Cohort C

	Minimum		Maximum		Mean		SD	
ATF4[c]	1.38		3.67		2.261		0.54529	
ATF5[c]	1.7		3.46		2.4779		0.47466	
CHOP[c]	4.04		7.05		5.21		0.86057	
	Affected	Unaffected	Affected	Unaffected	Affected	Unaffected	Affected	Unaffected
CN [c] SUV	3.2	3.6	12.2	13.3	6.759	7.1564	2.0229	2.0769
PP [c] SUV	2.7	2.7	7.4	7.8	4.6282	4.6744	1.2183	1.1811
AP [c] SUV	2.9	3.3	10.8	11.3	5.8051	6.4974	1.6894	1.7773

Supple Table 5. Correlation Analysis of Plasma UPR^{mt} Biomarkers with PET-CT SUVR values in Severe Side Brain Regions (CN, AP, PP) in cohort C

		Spearman's Rho (ρ)		Test Statistic (S)		P value			
		Affected	Unaffected	Affected	Unaffected	Affected	Unaffected		
CN	ATF4[c]	0.2401824	0.2310224	7507	7597.5	0.1408	0.1571		
	ATF5 [c]	/	/	/	/	/	/		
	CHOP [c]	-0.0531915	-0.06044958	10406	10477	0.7478	0.7147		
AP	ATF4[c]	0.1923817	0.190001	7979.3	8002.8	0.2407	0.2466		
	ATF5[c]	-0.0164582	/	10043	/	0.9208	/		
	CHOP[c]	0.04512078	-0.002278827	9434.2	/	0.785	/		
PP	ATF4 [c]	0.1901552	0.1487399	8001.3	8410.4	0.2463	0.3662		
	ATF5 [c]	/		/	9902.5	/	0.989		
	CHOP [c]	-0.1457606	0.05855817	11320	9301.4	0.3759	0.7233		
		Pearson's Cor index		Test Statistic (S)		P value		95% CI	
		Affected	Unaffected	Affected	Unaffected	Affected	Unaffected	Affected	Unaffected
CN	ATF5 [c]	-0.05929015	-0.0923214	-0.36128	-0.56398	0.7199	0.5762	[-0.3679244, 0.2611115]	[-0.3962948, 0.2298922]
AP		/	-0.0902530	/	-0.55124	/	0.5848	/	[-0.3945351, 0.2318668]
PP		0.1818175	0.1284685	1.1247	0.78797	0.268	0.4357	[-0.1418362, 0.4703521]	[-0.1949507, 0.4266901]

Upper Part: Spearman Analysis, Lower Part: Pearson Method.

Western blot full scans

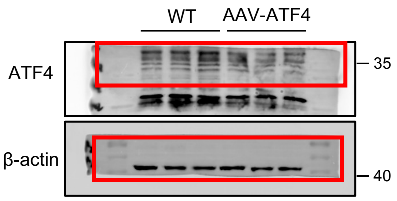

Fig. 1c

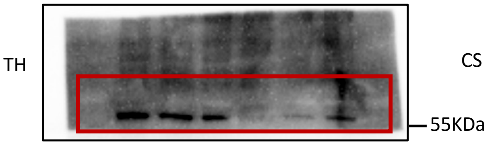

Revised Fig. 1m

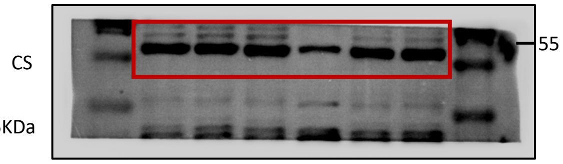

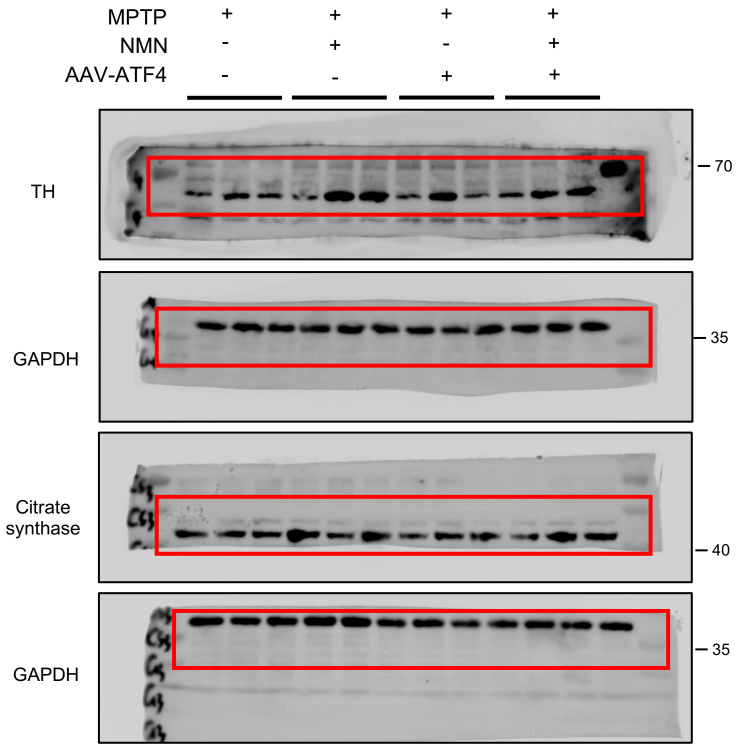

Revised Fig. 4f

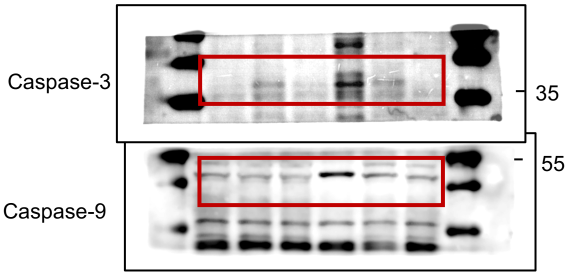

Revised Fig. 4h

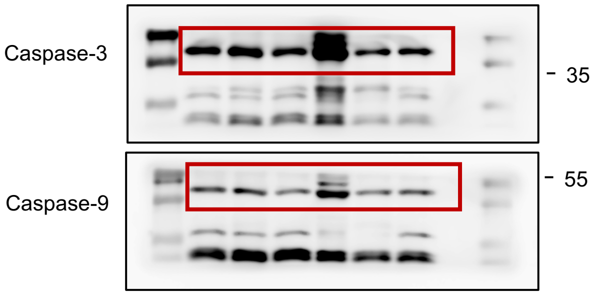

Revised Fig. 8f

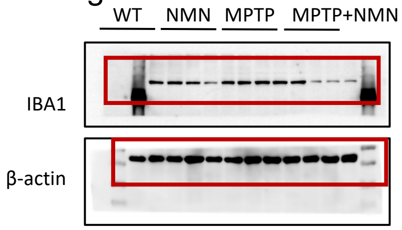

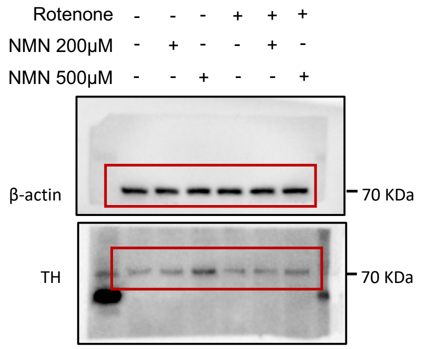

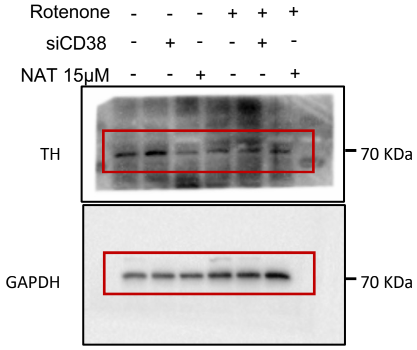

Revised Supplementary Figure 1e, f

Western blot full scans

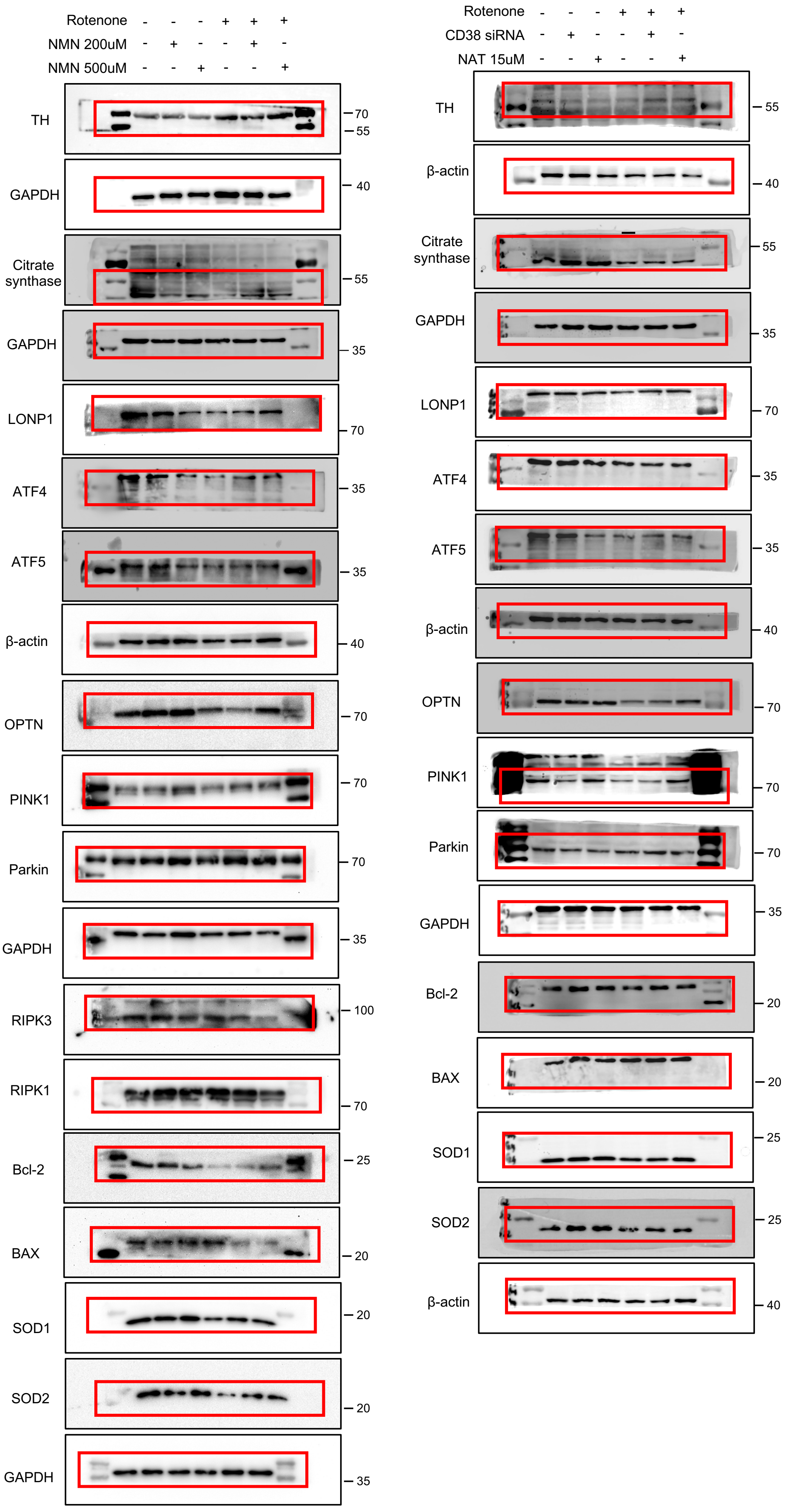

Western blot full scans

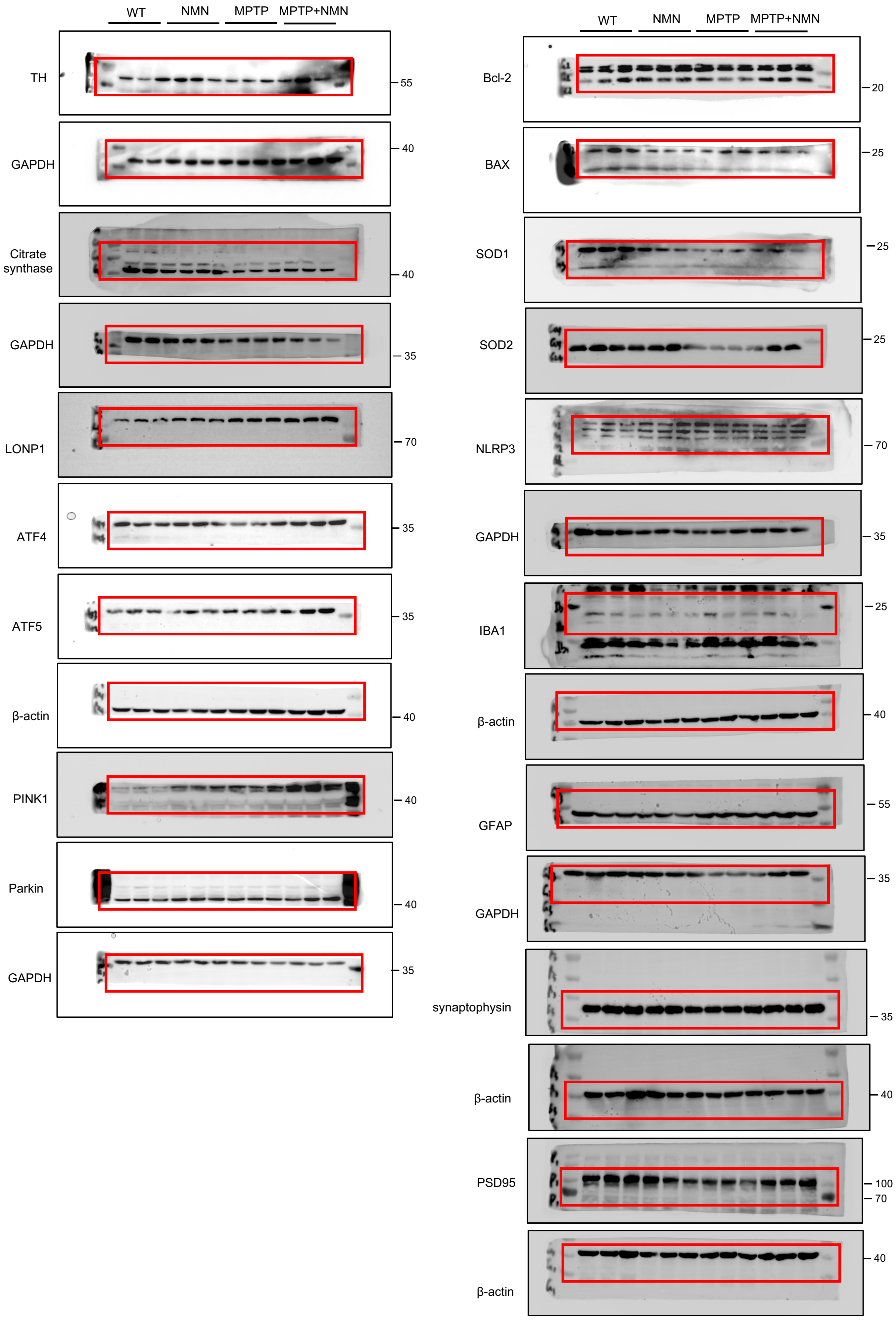

Supplement: Supplementary file 1 — Supporting Information [file ADVS-12-e08503-s001.pdf]
